# Does high workload reduce the quality of healthcare? Evidence from rural Senegal

**DOI:** 10.1016/j.jhealeco.2022.102600

**Published:** 2022-03

**Authors:** Roxanne Kovacs, Mylene Lagarde

**Affiliations:** a Department of Economics and Centre for Health Governance, University of Gothenburg, Vasagatan 1, Gothenburg, Sweden; b London School of Economics and Political Science, Department of Health Policy, Houghton Street, London, UK

**Keywords:** Workload, Quality of care, Standardised patients, Senegal, I10, I11, J45

## Abstract

There is a widely held perception that staff shortages in low and middle-income countries (LMICs) lead to excessive workloads, which in turn worsen the quality of healthcare. Yet there is little evidence supporting these claims. We use data from standardised patient visits in Senegal and determine the effect of workload on the quality of primary care by exploiting quasi-random variation in workload. We find that despite a lack of staff, average levels of workload are low. Even at times when workload is high, there is no evidence that provider effort or quality of care are significantly reduced. Our data indicate that providers operate below their production possibility frontier and have sufficient capacity to attend more patients without compromising quality. This contradicts the prevailing discourse that staff shortages are a key reason for poor quality primary care in LMICs and suggests that the origins likely lie elsewhere.

## 1 Introduction

According to the WHO, 90% of low-income countries suffer from a critical shortage of healthcare staff, defined as fewer than 4.4 qualified staff per 1000 population (WHO, 2011, 2016). There is a widely held perception that this lack of staff leads to an excessive workload, which in turn has a negative effect on the quality of healthcare delivered. For instance, the WHO note that in facilities without enough staff, providers “*may be forced to “cut corners” in order to cope with their workload. This may seriously reduce the quality of health services they provide*” (WHO, 2010; p.34). Similar concerns are expressed by UNICEF, noting that an “*overwhelming workload may lead to [providers] compromising the care of patients*” (Unicef, 2020; p.8). A high workload likely reduces both the motivation and ability of providers to offer appropriate care, which could perhaps explain the gap observed between what providers know how to do and what they do in practice (Das and Hammer, 2007; Leonard et al., 2007; Leonard and Masatu, 2010b; Mohanan et al., 2015; Gage et al., 2018). This paper investigates the widely held perceptions that *(i)* providers in low-income settings face an excessive workload and that *(ii)* a higher workload reduces the quality of healthcare delivered.

Conceptually, the relationship between workload and the quality of care is not straightforward. Central to this relationship is the potential trade-off that health workers may face between the number of patients seen and the quality of care provided to each one. At low levels of workload, an increase in the number of patients may have no effect on the quality of care, as providers have spare capacity to see new patients without having to reduce the time invested in each consultation. Insights from the psychology literature even suggest that increases in workload from very low levels may be beneficial, as increased activity may allow individuals to become more focused and attentive (Yerkes and Dodson, 1908). Yet, as workload increases, these beneficial effects likely disappear or are crowded out by the negative effect of increased stress, which can reduce cognitive performance (Bruggen, 2015). To serve an increasing volume of patients, it seems that providers will eventually have to reduce the quality offered to each one.

A number of characteristics of public-sector healthcare markets in low and middle-income countries (LMICs) may exacerbate the potential negative effect of workload on the quality of care. First, as providers generally have fixed consultation hours, the quantity-quality trade-off is particularly pronounced – as there is a mechanical relationship between the number of patients seen and the time dedicated to each one. Second, providers face limited costs for reducing effort and quality provided, as their salaries are not linked to performance,^1^ and providers in LMICs usually face virtually no legal or professional costs for providing inappropriate care (Björkman and Svensson, 2009).

Few studies in LMICs have explored the relationship between workload and the quality of care, due to two main empirical challenges. The first issue is that the quality of healthcare and workload are endogenous. If patients choose where and when to seek care, as they do for most non-urgent ailments, the demand for care, and therefore providers’ workload, is at least partly determined by the quality of care (or perceptions thereof). The second challenge lies in the difficulty of measuring quality of care and provider effort. Even in high-income settings, where electronic medical records are widely available, it is difficult, if not impossible, to ascertain whether doctors have provided an appropriate treatment to a patient, as their underlying condition is unknown. Studies in LMICs generally use observations of medical consultations to measure provider effort (Mæstad et al., 2010; Akachi and Kruk, 2017; Kruk et al., 2018). However, due to a Hawthorne effect,^2^ these measures likely over-estimate provider effort and could cancel out any potential effect of workload on effort.

In this paper, we overcome both of these empirical challenges. First, we use reliable measures of provider effort and quality of care obtained from an audit study, where five standardised patients (SPs) were sent to nearly 200 primary care facilities in rural Senegal. SPs are healthy individuals trained to present pre-specified medical cases to primary care providers, and subsequently record what happened during the consultation (Kwan et al., 2019). Data obtained from SP visits generate a rich set of measures of provider effort and quality of care and are often referred to as the “gold standard” (Luck and Peabody, 2002; Wiseman et al., 2019) – at least for the specific ailments portrayed. Second, to overcome the reverse causality issue, we exploit the fact that the timing of SP visits was quasi-random, as it was determined before fieldwork and based solely on logistic considerations. We capture whether SPs visited facilities during busy times – that is, when the number of patients attending was higher than on an average day for that facility.

Our study yields four main results. First, despite the supposed shortage of healthcare staff in rural Senegal, we find that workload in study facilities is low. Providers see between five and nine patients a day and spend no more than two hours doing consultations. Using secondary data, we show that workload is similarly low in other countries classified as having a “human resources for health crisis” (WHO, 2006a, 2016) such as DRC, Tanzania and Haiti – where providers in rural areas see less than four patients a day. Second, we find that the quality of primary care in study areas is low, with only 35% of patients correctly managed by providers. This is in line with a growing literature highlighting that patients in LMICs frequently receive incorrect medical advice and unnecessary treatments (Das et al., 2008; Mohanan et al., 2015; Sylvia et al., 2015; Das et al., 2016; Daniels et al., 2017). Third, although workload varies widely in rural Senegal, we find no evidence that quality of care is lower when workload increases. There is also no evidence that a higher volume of patients worsens process quality of care, measured by the actions completed by providers during the consultation, even though we cannot rule out a small, but statistically insignificant, decrease in consultation duration. Finally, our data suggest that the lack of a link between workload and healthcare quality is likely explained by providers operating well below their production possibility frontier. Our sense is that the *a priori* contradictory co-existence of severe staff shortages and low workload is partly explained by a *de facto* task shifting approach adopted by facilities whereby staff with lower qualifications take on roles that ought to be held by professional staff (doctors, nurses, midwives). Interestingly, although quality of care is low in the studied areas, our data do not suggest large quality gaps between professional cadres and unqualified staff.

Together with previous work from LMICs, our findings contradict the prevailing discourse that staff shortages and high workloads are key drivers of the poor quality of primary care in LMICs and suggest that other causes need further investigation.

This paper contributes to the empirical literature on workload and performance. There has been limited causal evidence on the relationship between workload and performance in healthcare. Most studies have been conducted in hospitals in high-income settings (Marks and Choi, 2019; Maibom et al., 2021). The majority of studies examine a correlation between workload and performance (Needleman et al., 2002; Duffield et al., 2011; Halpern, 2011; Ball et al., 2014; Snowden et al., 2017), and some examine a causal link and find mixed results (Hoe, forthcoming; Honeyford et al., 2018; Marks and Choi, 2019). In LMICs, most studies on workload are qualitative and document the perceptions of healthcare workers that high workload reduces the quality of care they are able to provide (Rouleau et al., 2012; Nagai et al., 2017; Jaeger et al., 2018). In addition, a small number of quantitative descriptive studies document the low levels of workload in LMICs (Das et al., 2020). We are only aware of one previous study that identifies a causal relationship between workload and the quality of healthcare in a low-income setting (Mæstad et al., 2010). Using an instrumental variable approach, Mæstad et al. (2010) investigate the causal link between provider effort (measured via overt observations of consultations) and workload in primary care facilities in Tanzania, finding no evidence that higher workload reduces quality of care. One concern with previous work is the reliance on overt observations of providers – which introduces a Hawthorne effect that could cancel out any relationship between workload and performance. We add to previous work in two main ways. First, we use a more reliable measure of provider effort, based on SPs, which avoid observation bias and provide detailed measures of the quality of history-taking and physical examination. Second, we provide a rich set of care quality measures. We test whether higher workload reduces the quality of case management in primary care (i.e. whether providers treat patients adequately). Whether patients are correctly managed is much more consequential for health outcomes than provider effort alone – which has been the focus of previous work.

This paper also sheds light on the implications of existing staff shortages in primary healthcare markets in LMICs. The idea that staff shortages lead to excessive workload and therefore poor-quality healthcare is popular amongst policymakers as well as healthcare providers (WHO, 2010b; Rouleau et al., 2012; Ahumuza et al., 2016; Nagai et al., 2017; Unicef, 2020). In addition, high workload is a potential reason for the gap between provider knowledge and practice (i.e. the know-do gap) identified in several LMICs (Das et al., 2008; Leonard and Masatu, 2010a; Lange et al., 2014; Mohanan et al., 2015). Adding to previous work in Tanzania (Mæstad et al., 2010) and India (Das et al., 2020) we show that in the Senegalese primary care sector, as well as in other LMICs, workload is generally low despite staff shortages. Low demand for care is likely due to a number of factors including the relatively high cost of seeking care due to user fees, time and travel costs (Gabrysch and Campbell, 2009; Bright et al., 2018). As a result, even at busy times, healthcare workers do not seem to have to compromise quality to see more patients. This finding is important to help dispel the common conception that low quality of primary care in LMICs is caused by high workload.

## 2 Data and measures

### 2.1 Study setting

This study was conducted in Senegal, a lower middle-income country in West-Africa. Data were collected in a representative sample of public primary healthcare facilities in four (out of 14) regions (Ziguinchor, Sédhiou, Tambacounda, Kédougou). These are rural and particularly disadvantaged areas in the country. The majority of households (70%) fall into in the two poorest national wealth quintiles and 38% of the population have not completed primary school (DHS, 2017). Use of primary care services is limited in study areas. Only 36% of children under five are fully vaccinated (DHS, 2017). For children with symptoms of fever, diarrhea and cough, respectively 51%, 45%, and 60% of children were taken to see a healthcare provider (DHS, 2017), even though healthcare is free of charge for children under five (UNICEF, 2013).

While consultations for children under five are free (Witter et al., 2010), all other patients have to pay for primary care consultations. Existing evidence suggests that user fees are significant in Senegal: out-of-pocket payments make up 56% of total health expenditure in the country (WB, 2016), in rural areas, households spend on average 10% of their income on healthcare and 6% of households incur catastrophic expenses (Massamba Séne and Cissé, 2015). According to a recent household survey, 56% of health spending goes towards buying medication, 38% is used to pay for tests and examinations and 6% goes towards consultation fees (ANSD, 2021). Data from our study suggest that adult patients in study areas pay on average 295 CFA (0.5 USD) for a consultation in a public health facility.^3^

There is a severe shortage of healthcare staff in study areas. The latest available staffing data suggest that, in 2011, there were only 56 doctors practicing in primary care facilities in study regions, serving over 3.3 million people (ANSD, 2012). The ratio of doctors, nurses and midwives per 1000 population falls far below the recommended WHO target of 4.4, as it is only 0.17 in Tambacounda, 0.18 in Sédhiou, 0.24 in Ziguinchor and 0.31 in Kédougou (ANSD, 2012). The Ministry of Health is trying to remedy the shortage of healthcare staff, particularly in rural areas, by training more healthcare providers. However, the limited capacity of educational institutions, low retention of staff in rural areas, as well as the high rate of emigration of healthcare providers (over one third of doctors emigrate to OECD countries) are key challenges (WHO, 2010a; OECD, 2021).

### 2.2 Data sources

Data were collected in health posts and health centres, which are the most common points of access for primary care. These facilities are staffed with nurses, midwives and unskilled providers (with paramedical or no medical training) who assure all types of consultations.

Data collection was organised into two phases. In the first phase (April-July 2016) enumerators visited a random sample of 198 primary care facilities, randomly drawn from the list of all 238 public primary healthcare facilities in study regions. During the visit, they administered surveys to collect information about the health facility and providers working there. Providers were also asked for their consent to be visited by SPs over the next six months.

In the second phase of data collection (November-December 2016), SPs attempted to visit the same health facilities. SPs presented five medical cases: dysentery in a child, stable angina, family planning, tuberculosis and asthma. The SP portraying a case of tuberculosis was randomised to only visit half of the facilities (*n* = 99), while the other half received a similar case that presented an experimental variation of the same scenario.^4^ SPs only attempted one visit to each facility, and ten attempts were unsuccessful, due poor weather, absenteeism of providers assuring a specific type of consultation or facilities being closed.^5^ In total, 881 consultations by SPs were done in 198 different facilities. As consultations are organised on a walk-in basis, not all providers seen by SPs were interviewed in the first phase of data collection. Overall, SPs saw 377 providers, information on socio-demographic characteristics is available for 366 of these (97%).^6^

Ethical approval for the study was obtained from the research ethics committees of the Senegalese Ministry of Health and the London School of Hygiene and Tropical Medicine.

### 2.3 Measuring provider effort and quality of care

We use SPs to measure the quality of case management as well as provider effort. SPs are healthy individuals who are trained to act out the symptoms of a specific medical case, visit healthcare workers undercover and subsequently record what happened during the consultation. SPs fill out a checklist immediately after the consultation, summarizing the questions asked and examinations performed by the provider, as well as the drugs or tests prescribed. SPs also record the duration of the consultation (by recording the time they entered and left the consultation room). The advantage of using SPs is that there is no observation bias, as SPs should not be distinguishable from real patients. In addition, SPs can be used to assess the quality of case management (i.e. the appropriateness of treatments received) – which, unlike provider effort alone, is relevant for health outcomes.

Based on previous studies and in collaboration with health professionals from Senegal and the UK, we developed five textbook medical cases: a mother bringing her 18-month-old child with symptoms of dysentery, a 55-year-old man complaining of chest pain (stable angina), a woman in her late 20 s with a history of migraines asking for a contraceptive method, a woman in her early 20 s complaining of breathing difficulties (asthma attacks) and a 35-year old man with persistent cough and fatigue, suggestive of tuberculosis (see Appendix 1 for a detailed description of each case).

The primary outcome of interest is the quality of care received, measured by the quality of case management. We use a binary variable indicating whether the treatments or recommendations given to patients were correct (i.e. clinically indicated) or incorrect. For instance, providers who referred the SP with symptoms indicative of stable angina to a higher-level facility, or performed and ECG, were coded as having provided managed the case correctly (see Appendix A1 for an overview of which treatments were considered clinically indicated for each case).

To capture our secondary outcome of interest, provider effort, we use two measures that are based on information provided by SPs in a questionnaire completed immediately after the consultation. In this questionnaire, each SP was asked to report the exact start and end time of the consultation. We used this to compute the duration of the consultation. Each SP also indicated whether the provider had asked them a series of questions and undertaken some physical examinations during the consultation. The list of recommended questions and physical examinations was specific to each case and we use it to capture the proportion of recommended actions performed by the provider.^7^

### 2.4 Measuring workload

We use the number of patients waiting to see the same provider at the facility when SPs arrived as a simple measure of workload – which we call patient load. Consultations in study facilities are conducted on a walk-in basis. When SPs arrived at a facility, they were generally given a consultation ticket (*ticket de consultation*) and assigned to a queue or waiting area for a provider assuring their type of consultation (for instance, antenatal care, pediatric care or adult care). We define patient load for provider p in facility f as the number of patients already waiting in line when SP *i* joined the queue ($PL_{ipf}$).

We also capture average levels of patient load, using data collected in the facility survey. Information on the number of outpatient (adult, child, ante-natal and family planning) consultations done monthly in each facility between January and March 2016 was extracted from facility registers. Based on these data, we estimate the average monthly number of consultations (${\bar{Consultations}}_{f})$. We also recorded the number of providers responsible for receiving patients, present in the facility on the day of the facility visit ($Staff_{f})$. Assuming that each provider works 20 days per month and does an equal share of consultations, we derive ${\bar{PL}}_{f}$ the number of consultations done per provider on an “average day” in facility *f*:

$${\bar{PL}}_{f}=\;\frac{{\bar{Consultations}}_{f}}{Staff_{f}\times \;20}$$

To compare the level of workload in study facilities to other settings in LMICs, we use data from the Service Provision Assessment (SPA) surveys, which provide information on service readiness and quality of care in a representative sample of public and private health facilities in selected countries. In some countries, SPA surveys contain information on consultation volumes as well as the type and number of staff working at the facility.^8^ We use data from surveys in DRC (2017), Senegal (2016), Bangladesh^9^ (2014), Tanzania (2014), Haiti (2013) and Malawi (2013) and compute, for each facility surveyed, the following measure of daily patient load per provider ($PL_{f}^{SPA}$):

$$PL_{f}^{SPA}=\frac{Consultations_{f}^{SPA}}{Personell_{f}^{SPA}\times \;20}$$

where $Consultations_{f}^{SPA}$ is the number of outpatient visits recorded in the previous calendar month in facility *f*, $Personell_{f}^{SPA}$ is the number of clinical staff listed as working in the facility (excluding laboratory staff, pharmacy staff, as well as other support staff such as cleaners, office help or community health workers). As before, we assume that providers work 20 days per month on average.

To provide a better comparison for our study, we compute national averages for primary healthcare facilities and disaggregate results by urban and rural areas. One limitation of the SPA surveys is that they do not measure absenteeism, and only record the number of staff assigned to the facility – rather than those who are present on the day of the visit. Hence, the measure of workload $PL_{f}^{SPA}$ is likely a lower bound estimate of workload. To account for this, we show levels of workload assuming absenteeism rates of 20% and 40%.^10^

### 2.5 Description of the analytical sample

To determine whether providers had suspected that SPs were not real patients, we contacted them by phone approximately four weeks after SP visits were conducted. We asked if providers had some suspicions about fake patients coming to their facility, and if so, to describe the patient case. Providers correctly suspected that in 64 consultations (7.2%) they had not received actual patients – most often their suspicions related to the fact that they had not seen the individuals before in the village. We exclude these consultations from the analysis, as not to bias estimates.

Table 1 shows the characteristics of facilities and health providers visited by the undetected SPs. The majority of healthcare facilities visited were health posts. Facilities were well equipped and stocked 78% of essential drugs and medical equipment. 52% of relevant medical guidelines were available and easily accessible in facilities. Facilities were located in remote areas as there were only 1.5 other facilities in a five-kilometer radius and the distance to the closest higher-level facility was 36 km on average. There were on average three healthcare providers registered as working in each facility. Each facility is intended to serve a target population determined by local health administrators, which was close to 7500 people, or 4200 people per provider.^11^

**Table 1:** Sample description.

	Mean	Std. Dev.
**Facility characteristics** (*n* = 195)		
Health post	0.93	0.26
% of drugs and equipment available	0.78	0.09
% of treatment guidelines available	0.52	0.26
Number of facilities in 5 km radius	1.45	2.6
Distance to closest higher-level facility (km)	36.48	34.82
Providers registered with facility	3.18	3.47
Size of catchment population	7495	6271
Catchment population per provider	4160	3596
**Provider characteristics** (*n* = 353)		
Male	0.48	0.50
Work experience (years)	9.30	8.52
Doctor	0.02	0.15
Nurse	0.26	0.44
Midwife	0.23	0.42
Nursing assistant	0.30	0.46
Other qualification	0.10	0.31
No qualification	0.08	0.27
Number of on-the-job training courses^(a)^	2.55	2.59
Provider born in local area (*n* = 241)	0.21	0.4
Salary (in 1000 FCFA) (*n* = 355)	134	84
My salary is satisfactory given my work	0.13	0.34
I am unhappy with my workload	0.44	0.50

*Note:* Data on facility characteristics are based on interviews with facility managers conducted in April-July 2016 during the first round of data collection (before SP visits). Data on providers are based on interviews conducted at the same time with the providers present at the facility on the day of the visit. Undetected SP visits took place in 195 facilities. Undetected SPs saw 364 providers but information on provider characteristics are available for only 353 of them. Data on salary are only available for *n* = 343 providers, data on place of birth is only available for *n* = 229 providers, data on work and workload satisfaction are available for *n* = 220 providers. (a) Providers were asked about on the job training courses for tuberculosis, family planning, non-communicable adult diseases and integrated management of childhood illnesses.

Providers seen by SPs were equally likely to be male or female, and they had on average nine years of work experience. Only 2% of providers who consulted SPs were doctors and half were nurses and midwives. 48% of the SPs were seen by nursing assistants, providers with other qualifications (e.g. training nurse) or individuals with no medical qualification. Providers completed on average three on-the-job training courses in the past year. Only 20% of providers were born in the area where they were working – reflecting the central assignment of staff to facilities by the Ministry of Health. Providers earned on average 134,000 FCFA (roughly €200) per month. In the provider survey, only 13% indicated feeling that they earn an adequate salary and 44% reported that they were unhappy about their workload.

Table 2 shows descriptive statistics for SP visits. SPs waited on average one hour to be seen by providers. In terms of provider effort, providers completed about a third of recommended actions (relevant history questions asked, physical examinations performed) and a consultation latest 13 min on average – between 10 min for the child with dysentery and 20 min for the family planning case. These levels of effort translated into overall poor quality of care. The probability that a patient was correctly managed by the provider was 35% and ranged from 19% for the family planning case to 57% for the patient displaying signs of tuberculosis. 67% of the drugs prescribed by providers were unnecessary and 7% were harmful.

**Table 2:** Quality of care during SP visits.

	All SP cases	Dysentery	Angina	Family Planning	Tuberculosis	Asthma
Waiting time (min.)	54.24	56.02	56.53	46.51	59.61	54.84
% of SPs seen by professional staff	0.49	0.40	0.44	0.67	0.45	0.49
% of relevant actions done	0.31	0.21	0.30	0.38	0.33	0.34
Duration of consultation (min.)	12.62	9.52	10.17	20.03	10.34	12.29
Any drugs prescribed	0.90	0.99	0.79	–	0.78	0.90
Number of drugs prescribed	2.20	2.96	1.43	–	2.18	2.24
Any unnecessary treatment	0.67	0.45	0.79	–	0.71	0.76
Any harmful treatment	0.07	0.01	0.22	–	0.00	0.02
Correct case management	0.35	0.27	0.39	0.19	0.57	0.43
						
Observations	817	182	185	173	89	188

*Note:* Data come from the questionnaires filled by SPs immediately after their visits, which occurred in November-December 2016. The sample only includes SPs who were not detected by providers. FP stands for family planning and TB stands for tuberculosis. Professional staff refers to doctors, nurses and midwives – available for *n* = 797 consultations. Relevant actions done refers to the proportion of relevant history questions asked and physical examinations performed by providers. Relevant actions refer to a list of questions and physical examinations included in a questionnaire completed by SPs after the consultation. This list was established by local and international experts to reflect what was considered relevant investigation a provider should be doing. Unnecessary and harmful drugs are defined for each case in Appendix A1.

## 3 Identification strategy

### 3.1 As-if-random variation in workload

To try and establish a causal relationship between workload and the quality of care in Senegal, we construct a measure of how busy a health facility was when a SP arrived. We combine information on patient load (i.e. the number of patients waiting in line to see the provider *p* when SP *i* arrived at the facility, $PL_{ipf}$) with data collected from facility registers (${\bar{PL}}_{f}$ or the number of patients seen per a provider on an “average day” in facility *f*). Our primary measure of busyness is a dummy variable equal to one when the number of patients queuing at the facility to see a given provider when the SP arrived exceeds the number of patients seen by a provider on an “average day”, i.e. if $PL_{ipf}>{\bar{PL}}_{f}$ – in other words, when the patient queue for provider *p* is particularly long.^12^

Because of the study design, it is unlikely that our measure of busyness is endogenous to the quality of care. SPs visited each facility based on a strict schedule devised before commencing fieldwork. SPs always visited facilities on two consecutive days. The research team planned the visits based solely on logistical considerations and had no means to predict whether a given day would be busy or not, and whether the time at which the patient would arrive was going to be busy or not. During fieldwork, the research team was in daily contact with SPs to ensure that facilities were being visited in the pre-specified order. For all but three consultations (0.3%), SPs complied with this schedule and visited facilities as planned. As a result, whether or not SPs arrived at the healthcare facility at a busy time was as-if random.

**Fig. 1:**
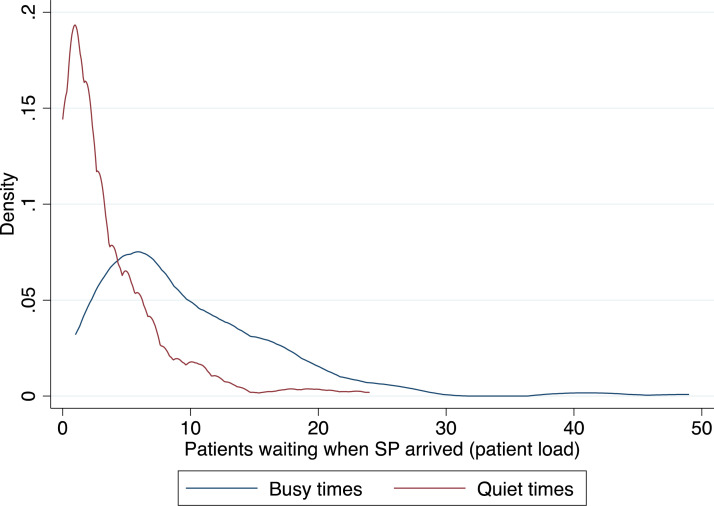
Patient load during busy and quiet times.

*Note:* The figure shows the distribution of patients waiting to be seen by the same provider when SPs arrived, for busy and quite times. An SP consultation is defined as having occurred during a busy time when the number of patients waiting to be seen by a provider when the SP arrived exceeded the number of patients seen on an “average” day by a provider in that facility.

In Appendix Table A1 we show that there are no major differences in facilities and providers visited during busy or quiet times. The only difference for facilities is that the proportion of health posts is higher amongst quiet facilities compared to busy ones,^13^ which is not unexpected given that health centres are larger facilities which tend to have greater patient flows. We also find that providers considered as busy (that is, if at least one of the SP consultations they conducted was coded as busy) completed 0.7 more on-the-job training courses than ‘quiet’ providers. The general lack of significant differences between busy and quite facilities and providers supports the argument that busyness is quasi random. Overall, 23% of consultations were conducted during busy times. Fig. 1 shows the distribution of patient load for busy and quiet times. During busy times, patient load was 1.1 SD higher than during quiet times (9.8 compared to 3.5 patients).

### 3.2 Econometric specifications

We first examine the correlation between patient load (i.e. the number of patients waiting to be seen on the day SPs arrived) and the quality of care. All analyses are conducted at the consultation level (standard errors are clustered by health facility). We first capture the simple association between patient load and the quality of care. We estimate the following OLS regressions:

(1) $$Y_{ipf}=\beta_{0}+\beta_{1}PL_{ipf}+\beta_{2}X_{p}+\beta_{3}Z_{f}+\delta_{c}+\delta_{a}+\delta_{r}+\delta_{d}+\lambda_{f}+\theta_{dow}+e_{ipf}$$

The dependent variable $Y_{ipf}$ captures the three outcomes of interest (the proportion of relevant questions asked and examinations done, the duration of the consultation and correct case management) for consultation i done by provider p in facilityf. The measure of patient load, $PL_{ipf}$ is measured at the consultation level and refers to the number of patients waiting in line at a facility, to be seen by provider *p*, when SP *i* arrived to consult the same provider. We control for provider $\left(X_{p}\right)$ and facility characteristics $\left(Z_{f}\right)$. We also include SP case ($\delta_{c})$, SP individual/actor $\left(\delta_{a}\right)$, region ($\delta_{r})$ and district ($\delta_{d})$ fixed effects. As the endogenous relationship between workload and the quality of care arguably arises due to differences between facilities – because patients might prefer to attend facilities where quality of care is higher – we include facility fixed effects ($\lambda_{f}$). To account for the possibility that patients may know that facilities are busier on certain days, we also include day of the week fixed effects ($\theta_{dow})$.

Next, to try and establish a causal relationship between workload and the quality of care, we use a measure of whether consultations occurred during a time when facilities were busier than usual. Whether or not SPs attend facilities during a busy time is as-if-random due to the study design, meaning that there should be no concerns about reverse causality. We estimate the following OLS regressions:

(2) $$Y_{ipf}=\beta_{0}+\beta_{1}Busy_{ipf}+\beta_{2}X_{p}+\beta_{3}Z_{f}+\delta_{c}+\delta_{a}+\delta_{r}+\delta_{d}+\lambda_{f}+\theta_{dow}+e_{ipf}$$

where $Busy_{ipf}$ is a variable indicating how busy provider p in facility *f* is when SP *i* arrives. As indicated in the previous section, our preferred measure of busyness is a dummy variable which indicates whether the queue of patients waiting to be seen by provider *p* is unusually long, such that $PL_{ipf}>{\bar{PL}}_{f}$. We also use alternative measures of busyness, such as a continuous measure, which divides patient load when the SP arrived ($PL_{ipf}$) by the average patient load in the facility (${\bar{PL}}_{f}$). We then use that continuous measure to compute quintiles and deciles in the distribution of busyness.

## 4 Results

### 4.1 How high is provider workload?

**Fig. 2:**
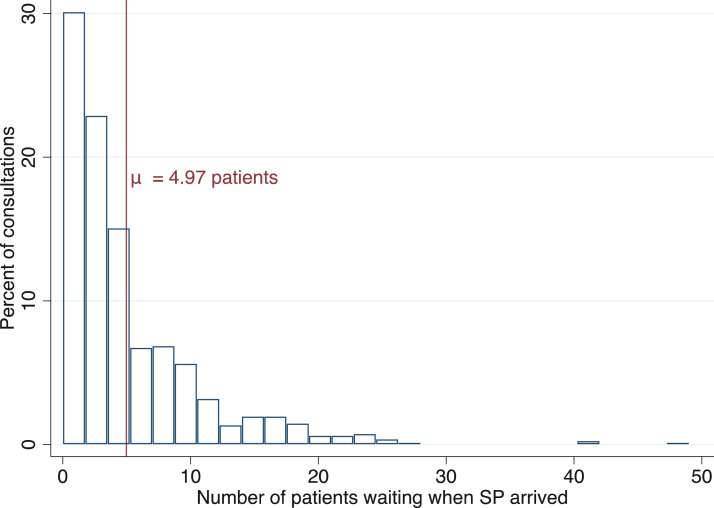
Variation in patient load (based on SPs).

*Notes:* The figure shows the distribution of the number of patients waiting to be seen at the facility when SP arrived. The standard deviation is 5.7.

**Fig. 3:**
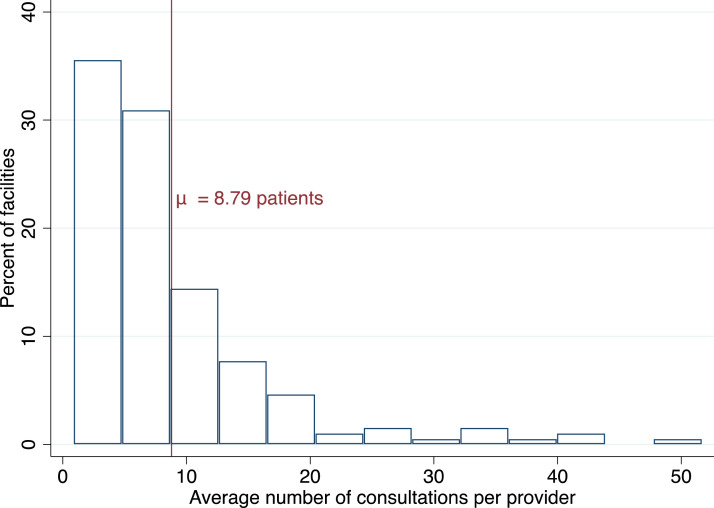
Variation in the average number of consultations (based on facility registers).

*Notes:* The figure shows the distribution of the average number of consultations done per provider per day, based on facility registers. The standard deviation is 8.0.

According to the WHO and national policy documents, there is a severe shortage of healthcare staff in Senegal, and in particular in study areas. However, contrary to a prevailing perception, the workload of healthcare providers appears to be low on average in study areas. We use two sources of data. First, as shown in Fig. 2, data from SPs suggests that five patients were on average waiting to be seen by a provider when SPs arrived in study facilities (SD = 5.7). Second, as shown in Fig. 3, administrative data collected in the first phase suggest that the average patient load in study facilities is nine patients per provider per day (SD = 8.0). Whilst average levels of workload are low, there is considerable variation. Overall, 15% of SP consultations were conducted with no other patients waiting to be seen whilst during the busiest 2% of consultations between 21 and 49 patients were waiting to be seen.

These low levels of workload do not appear to be unique to the study setting. Table 3 shows the number of consultations done per provider per day in primary care facilities in Bangladesh, DRC, Haiti, Malawi, Senegal and Tanzania. According to the WHO, all of these countries suffer from a severe shortage of healthcare staff (WHO, 2016). Yet the data suggest that workload is low in many of these settings. Providers in the DRC, Tanzania and Haiti see less than four patients a day. Nationally representative data from Senegal indicate that provider see roughly eight patients a day (highly similar to what we find in the study setting). Providers in Bangladesh see between 22 and 24 patients a day, which is considerably higher – however, if we assume that consultations last 10 min each, then providers would nonetheless spend just over 3.5 h a day doing consultations. As data from the SPA do not adjust for provider absenteeism, it is likely that these are lower-bound estimates of provider workload. However, even if we assume that staff are absent 40% of the time, providers in DRC, Tanzania and Haiti still see less than six patients per day, compared to 13.6 consultations in Senegal.

**Table 3:** Number of patients seen per provider per day in primary care facilities in LMICs.

	Unadjusted	Assuming 20% absenteeism	Assuming 40% absenteeism
**DRC (*N*** = 731)			
Primary care facilities	2.9	3.6	4.8
Rural	3.2	4.0	5.4
Urban	1.3	1.6	2.2
**Tanzania (*N*** = 922)			
Primary care facilities	3.4	4.2	5.6
Rural	3.6	4.5	6.0
Urban	2.7	3.4	4.5
**Haiti (*N*** == 857)			
Primary care facilities	3.4	4.2	5.6
Rural	3.5	4.4	5.8
Urban	3.1	3.9	5.2
**Senegal (*N*** = 317)			
Primary care facilities	8.1	10.2	13.6
Rural	9.2	11.4	15.3
Urban	6.4	8.0	10.7
**Bangladesh (*N*** = 935)			
Primary care facilities	21.5	26.8	35.8
Rural	23.5	29.3	39.1
Urban	15.8	19.7	26.3
**Malawi (*N*** = 738)			
Primary care facilities	24.5	30.6	40.8
Rural	28.0	35.0	46.7
Urban	14.7	18.3	24.4

*Note:* Data come from SPA surveys: DRC (2017), Tanzania (2014), Haiti (2013), Senegal (2016), Bangladesh (2014) and Malawi (2013). The table reports the average number of consultations done per provider per day, based on the number of monthly consultations divided by the number of clinical staff who are assumed to be working 20 days per month. As SPA data give no indication of absenteeism rates, we compute workload levels assuming 20% and 40% staff absenteeism.

Interestingly, the SPA data show that provider workload is higher in rural primary care facilities compared to urban facilities. This is the case because urban facilities are generally much better staffed but do not conduct proportionally more consultations than rural facilities. However, in the absence of reliable absenteeism rates, which potentially differ across areas, the actual differences in workload between rural and urban areas are unclear.

### 4.2 Workload and the quality of care

Although patient load is low on average, there are large variations in the volume of patients waiting to be seen by one provider. We use this variation to explore the relationship between workload and quality of care, with a view to isolate the causal effect of workload.

Panel A of Table 4 examines the simple association between patient load and provider effort and the quality of care (based on Eq. (1)). Overall, there is no evidence suggesting that patient load on the day of the consultation is associated with provider effort (proportion of items done or the duration of the consultation). Perhaps due to the absence of a link to provider effort, we find no evidence suggesting that patient load is associated with the quality of case management. Coefficients in all models are precisely estimated and very close to zero. To account for the possibility that the relationship between workload and the quality of care might be non-linear and for instance take an inverted U-shape we also add a quadratic term in Table A2 in the Appendix, and do not find that this alters our results.

**Table 4:** Workload, provider effort and the quality of healthcare.

	% relevant actions		Duration		Correct management	
	(1)	(2)	(3)	(4)	(5)	(6)
**Panel A: Patient load**						
Patient load	−0.001	−0.001	−0.019	−0.030	0.003	0.001
	(0.002)	(0.002)	(0.079)	(0.084)	(0.006)	(0.006)
						
Facility and provider controls	No	Yes	No	Yes	No	Yes
						
Clusters (facilities)	195	195	195	195	195	195
Observations (consultations)	817	797	817	797	817	797
R-squared	0.530	0.537	0.542	0.552	0.315	0.328
**Panel B: Busyness (quasi-random variation)**						
Busy time	−0.005	−0.001	−1.433	−1.538	−0.095	−0.107
	(0.019)	(0.020)	(0.863)	(0.909)	(0.068)	(0.073)
						
Facility and provider controls	No	Yes	No	Yes	No	Yes
						
Clusters (facilities)	194	194	194	194	194	194
Observations (consultations)	816	796	816	796	816	796
R-squared	0.529	0.537	0.544	0.554	0.316	0.330
Outcome mean	0.308		12.619		0.351	

*Notes:* All models show coefficients from linear (OLS) regressions. The dependent variables are: (1–2) the proportion of relevant actions (history questions and physical examinations) done by providers, (3–4) the duration of the consultation in minutes and (5–6) the probability of correct case management. In Panel A, dependent variables are regressed on patient load, which is the number of patients waiting in line to be seen by the same provider when an SP arrived at the facility (Eq. (1)). In Panel B, dependent variables are regressed on a dummy variable indicating whether an SP consultation was conducted during a busy time – i.e. when the number of patients waiting to see the same provider when the SP arrived at the facility exceeded the number of patients seen on an “average” day for that facility (Eq. (2)). We lack data on busyness for one SP consultation, as data on “average” consultation volumes could not be collected in the facility where this visit occurred. All models control for region, district, facility, day-of-the-week, SP case and SP actor fixed effects, as well as the time of day (i.e. the hour) the visit occurred. Facility characteristics (facility type, availability of essential drugs and equipment, distance to closest higher-level facility, availability of essential treatment guidelines, number of facilities in 5 km radius). Provider characteristics (gender, work experience, on-the-job training, level of education). Standard errors clustered at the health facility level in parentheses. ***p<0.01, **p<0.05, * *p*<0.1.

Panel B of Table 4 examines whether provider effort and the quality of care are lower at times when facilities are busy (based on Eq. (2)). As our measure of busyness is quasi-random, this specification largely addresses concerns about endogeneity. As shown in Panel B of Table 4, we find no evidence that provider effort or the quality of case management differ significantly when workload is particularly high (i.e. during busy times). We cannot rule out that consultations are somewhat shorter during busier times, as (non-significant) coefficients indicate that consultations might be 90 seconds shorter – which corresponds to 0.2 SD or 13%. However, these would traditionally be considered small effect sizes.^14^ Furthermore, we find no effect of how busy a provider is and the quality of care they provider. For the two key outcomes of quality of care, the proportion of relevant actions done as well as the clinical quality of care, coefficients are not significant and close to zero.

As shown in Appendix 2, the results remain unchanged when alternative measures of busyness are used. We find no evidence for an effect of busyness on the quality of healthcare when a continuous measure is used (Table A3), or a measure that captures quintiles or deciles of busyness (Table A4 and A5). In addition, to account for a non-linear relationship, we use different thresholds for determining whether a day was busy. In Table A6, we code consultations as busy when patient load was at least 1.25 times, 1.5 times, 1.75 times, twice or three times the workload on an average day. Our results remain largely unchanged when different thresholds are used and we fail to find a significant effect on the quality of care even at very high levels of workload.

### 4.3 Slack capacity despite staff shortages

Why do we not find evidence for a link between workload and quality of care? One explanation would be that there is considerable slack capacity in health facilities and that providers do not need to reduce the level of effort they invest to see more patients. Similar to providers in other LMICs, providers in the study setting see between five and nine patients a day. If we assume that an average consultation lasts 13 min (as suggested by SP data), this would imply that healthcare providers spend between 65 and 117 min a day consulting with patients. Even though providers also have administrative responsibilities (filling out registers, accounting or ordering drugs), need to attend on-the-job trainings and take part in community engagement activities – this does suggest that there is considerable slack capacity in healthcare facilities.

**Fig. 4:**
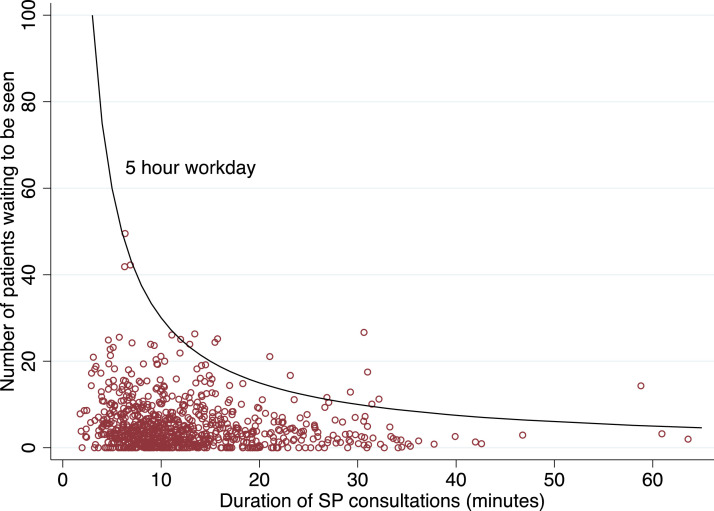
Patient load and consultation duration.

*Notes:* The red circles show, for each SP visit, the number of patients waiting to be seen when the SP arrived and the corresponding duration of the SP consultation. The black reference line indicates, for each consultation duration, the number of patients that a provider would have to see for that provider to spend five hours a day consulting patients. The figure excludes one consultation with a duration of one minute to avoid skewing the y-axis.

Fig. 4 plots the number of patients waiting to be seen when SPs arrived at the facilities against the duration of SP consultations (followin Das et al., 2020). The black reference line represents the production possibility frontier and shows the number of patients that would be required for each duration so that providers spend five hours a day consulting patients – which would leave ample time for administrative and other duties. The aim here is not to show how many staffing hours would be needed to provide high quality care, but rather to examine how much of their day providers spend with patients.^15^ If providers were working at capacity, we would expect most observations to lie on (or around) the production possibility frontier for a generous five hour working day. However, as shown below, virtually all consultations (99%) are shorter than what would be required to fill a five-hour working day for a given patient load – well below the frontier.

How can we reconcile the finding that staff have spare capacity with claims that Senegal, and many other LMICs, suffers from a “severe shortage of staff” (WHO, 2016)? Two factors help resolve these apparently contradictory findings. First, the definition of staff shortages used by the WHO or governments is needs-based in the sense that staffing numbers are determined based on what is thought to be required to meet specific health care delivery targets, based on the needs of the target population (Scheffler and Fulton, 2013). Yet in practice, there can be a discrepancy between the number of staff calculated in this needs-based approach and the actual demand of healthcare in some areas. In some settings, the number of staff required by the staffing norms may not be required to meet the demand of healthcare addressed to the facility. This gap may reflect the difference between the estimated needs of the population and the expressed demand. In turn, this points to the potentially detrimental role of user fees, which may limit access to healthcare services in a context like rural Senegal.

Second, as highlighted in Table 2, the type of health workers who receive patients are often not the professional cadres (doctors, nurses and midwives) included in the calculation of staffing norms, but are often individuals without sufficient training. It seems that facilities have adopted a task shifting solution in the rural areas studied, where providers without qualification or very low-levels of training take on roles that should be held by professional cadres. This explains why, despite a lack of professional staff, providers see a modest number of patients. Interestingly, as shown in Table A7 in the Appendix, we find only small differences in effort and quality of care provided by professional cadres and less qualified staff, although this warrants further exploration in larger samples.

### 4.4 Robustness checks

In this section, we address several potential concerns with the analysis and check the robustness of our results to alternative specifications.

First, to account for possible measurement error in our measure of busyness, we use the weekday of the consultation as an instrument for patient load. We exploit that patient load is higher Mondays than other days. This is likely due to facilities being open only from Monday to Friday, meaning that Monday is the first opportunity to seek care for patients who fall ill over the weekend. As discussed in detail in Appendix 3, we find a significant link between Mondays and workload in the first stage, although Monday appears to be a weak instrument. The second stage of the IV strategy confirm our main results, as we fail to find a significant effect of workload on provider effort and quality of care provided. Nevertheless, we interpret the IV results with caution, given that this is a predictable source of variation for providers and that Mondays could potentially influence workload through channels other than workload (e.g. motivation). Nevertheless, this alternative strategy also does not suggest a link between workload and the quality of primary care in rural Senegal.

Second, one might argue that we should only expect to see an association between workload and the quality of case management for the subset of providers who have sufficient knowledge to correctly treat patients. Arguably, providers without relevant knowledge would be unable to correctly manage patients regardless of the amount of time at their disposal. To test this, we interact workload with provider knowledge, captured in a clinical knowledge test described in Appendix 4. As shown in Table A12 in the Appendix, we do not find evidence that provider knowledge moderates the association between workload and the quality of case management.

A third concern is that our measures of patient load and busyness may be particularly prone to measurement error in larger clinics. This is because while patients queuing in front of a consultation room may be a good indicator of workload per provider in the small facilities in our sample (health posts, i.e. 93% of facilities), where one midwife generally conducts antenatal care and family planning consultations in one room and a nurse conducts all other visits in another room, in larger facilities patients could be queuing for more than one provider, or switch between queues. Hence in larger facilities (health centres), where more than one provider assures the same type of consultations, queues might be a less accurate measure of workload per provider. We therefore re-run the analysis excluding larger facilities. As shown in Table A13 in the Appendix, our results are practically identical when only health posts are included.

Fourth, we cluster standard errors at a lower level (i.e., at the provider level instead of the facility level). As shown in Table A14 in the Appendix, this does not alter our results.

Fifth, we use waiting times as an alternative measure of patient load. The results shown in Table A15 in Appendix confirm our main results. We find no evidence for an association between waiting time and measures of provider effort (relevant actions done, duration of the consultation) or quality of care.

Finally, we address the concern that our overall null results could be due to heterogeneity in workload between facilities, as facilities with high and low average patient load might respond differently to busy times. We split the sample in two, with facilities in the top 75th percentile of average patient load $\left({\bar{PL}}_{f}\right)$ coded as having “high” patient load and those below classified as having “low” patient load. Table A16 shows the results of the analysis conducted in both sub-samples. Overall, the absence of link between workload and busyness and provider performance remains the same in both groups. That being said, for both the duration of the consultation as well as the quality of case management, coefficients on busyness are higher for the sub-sample of facilities with low average patient load (although not significant), than for the sub-sample of facilities with high average patient load. This could suggest that a higher workload could have a worse effect in facilities that are less used to dealing with large volumes of patients, potentially because staff are less prepared or become more stressed and overwhelmed when this happens.

## 5 Discussion and conclusion

There is a widely held perception among policymakers and healthcare providers that staff shortages lead to an excessive workload, which negatively affects the quality of care provided in LMICs (WHO, 2010b; Unicef, 2020). We test both of these assumptions using data from Senegal – which, according to the WHO, suffers from a critical shortage of staff. Contrary to what one might have expected, workload is low in study areas, as primary care providers see between five and nine patients a day on average – although this varies widely, with zero to 49 patients queuing to see a provider. Even though it is plausible that the quality of care should be lower on days where workload is higher, this is not what our result are showing. We cannot rule out that a higher workload leads to a small (though statistically insignificant) decrease in the duration of medical consultations. However, we find no evidence suggesting that higher workload reduces the quality of case management or the proportion of relevant actions done by providers. We are able to examine threshold effects and fail to find a significant association between workload and healthcare quality even at very high levels of workload.

Our findings are in line with the only previous study we are aware of that tried to establish a causal link between workload and the quality of care in a low-income setting (Mæstad et al., 2010). Mæstad et al. (2010) find no evidence to suggest that workload affects provider performance in primary care facilities in Tanzania. The literature in high-income settings – where providers are highly skilled and operate in a much more regulated health system – is more mixed. Some studies find small or no effects of overcrowding in hospitals (Marks and Choi, 2019; Maibom et al., 2021) or primary care (Harris et al., 2019). Other studies in hospital settings find positive effects of nurse staffing on patient outcomes (Needleman et al., 2002; Ball et al., 2014) or negative effects of overcrowding on quality measure such as readmission (Hoe, forthcoming).

A plausible explanation for our findings is that providers are working below their production possibility frontier and have considerable slack capacity to see more patients. Providers spent no more than two hours doing consultations and 99% of SP consultations are shorter than what would be required to fill a five-hour working day for a given patient load. We show data from several other LMICs with severe shortages of staff to highlight that such low levels of workload are not unique to the study setting. There are two reasons why low levels of provider workload can coexist with shortages of professional staff. On the one hand, claims about staff shortages are based on needs-based approaches which do not necessarily reflect the effective demand faced by facilities. On the other hand, we demonstrate that facilities in our sample adopt a task shifting strategy where staff with low levels of qualification, or no formal qualification, take on the role of professional cadres.

This study has several limitations. First, even though we capture whether providers are busy consulting patients, we have no information on other activities – such as administrative duties. Whilst it would have been informative to control for the amount of time providers spend on these activities on the day SP consultations were conducted, such data are challenging to collect without introducing an observation bias. Second, the sample size in the study is modest (*n* = 817 consultations). Although it is possible that we are unable to detect a significant relationship between workload and the quality of case management for this reason, coefficients are generally close to zero, meaning that any effect would be close to zero.

Several considerations should caution against extrapolating our results beyond the study setting. To begin, we explore how providers respond to an idiosyncratic variation in workload on a day-to-day basis, or within a day. However, it may be challenging for providers to adjust their effort and performance to such variations, which could explain the null results we find. Providers may adapt to more systemic, longer-term variations in workload, and meet target levels of effort (e.g. number of consultations, duration of consultation) based on usual or expected levels of workload. Furthermore, we only focus on primary care facilities, in rural settings. It is unclear to what degree our results would hold in hospital settings, or for maternity care, where longer waiting times caused by higher workload are likely more detrimental to patient outcomes than in primary care. It is also unclear to what degree findings can be generalised beyond the specific conditions portrayed by SPs and the rural areas studied here, where personnel are likely to be less skilled and motivated (Lehmann et al., 2008). Further studies are needed to determine whether results hold for other conditions, or in settings where workload is higher.

In line with previous work from Tanzania (Mæstad et al., 2010) and India (Das et al., 2020), this paper challenges widely held beliefs about the implications of staff shortages in primary care facilities in LMICs. We show that in rural Senegal, higher workload does not translate into lower quality of care, likely because workload is low despite supposed staff shortages, meaning that healthcare workers still have a lot of spare capacity to receive patients. This finding has important implications for policies aiming to improve the quality of primary healthcare. The prevailing discourse has long been that structural weaknesses, such as a lack of drugs, medical supplies and staff, are to blame for poor quality of care in LMICs. Together with a growing body of evidence, this paper shows that higher workloads do not necessarily lead to worse quality of care. A future agenda of research has to go beyond these traditional explanations and explore other drivers of poor provider performance – for instance, cognitive biases might play an important role in limiting the performance of primary care providers in LMICs (Kovacs et al., 2020). In the absence of such evidence, governments run the risk of wasting limited resources on interventions that do not lead to improvements in health outcomes.

## Funding

This research was funded by ESRC/MRC/DfID/Wellcome Trust grant MR/M014681/1 as well as via a Wellcome Research Fellowships in Humanities and Social Science 219744/Z/19/Z.

## CRediT authorship contribution statement

**Roxanne Kovacs:** Conceptualization, Data curation, Formal analysis, Writing – original draft. **Mylene Lagarde:** Funding acquisition, Supervision, Writing – review & editing.

## Declaration of Competing Interest

None to declare.

## Appendix Supplementary materials

Image, application 1

## Footnotes

1 Even in systems where patients pay for the consultations and drugs, healthcare workers typically receive a very limited fraction of the money raised from user charges as salary.

2 Where effort increases in response to being observed (Leonard and Masatu, 2010b).

3 This is equal to roughly a third of daily consumption expenditure, which is estimated at 1,014 CFA on average in rural areas (ANSD, 2021).

4 This experimental variation was part of an embedded study which tested the effect of information presented by patients on the quality of care received. The results are presented elsewhere (Kovacs, Lagarde and Cairns, forthcoming).

5 There were five unsuccessful visits for the child with symptoms of dysentery, two for the stable angina case, two for the tuberculosis case and one for the family planning case.

6 For 125 of these 366 providers, information on socio-demographic characteristics was obtained through a telephone survey conducted after SP visits in March 2017.

7 SP cases were chosen to ensure that no invasive physical examinations would be required for correct case management. Hence, providers were able to conduct all relevant physical examinations listed on the checklist in Appendix A1 during the consultation.

8 SPA surveys only contain questions about consultation volumes since 2012. We included countries where SPA surveys were implemented after 2012. An SPA survey was conducted in Nepal in 2015, but the questionnaire did not include questions about monthly consultation volumes.

9 SPA data from Bangladesh only include public facilities.

10 These absenteeism rates were selected based on data from the Service Delivery Indicators project, which provide information on absenteeism in public health facilities for selected countries, including Senegal and Tanzania. Data from Senegal suggest that 20% of providers in rural and urban areas were absent during a random spot check (WB, 2012a). In Tanzania, 17% were absent in rural areas, compared to 33% in urban areas (WB, 2012b).

11 Note that these officially defined target populations refer to the number of people facilities are intended to serve, which can be larger or smaller than the population they actually serve.

12 We lack data on busyness for one SP consultation as data on usual consultation volumes ${\bar{PL}}_{f}$ could not be collected for the facility where this visit occurred.

13 A busy facility is defined as a facility where at least one SP visit occurred during a busy time (see definition in Section 3.1).

14 We also find no evidence of a significant reduction in the duration of the consultation on busy days in any of the alternative specifications used.

15 In the study setting consultations last 13 minutes on average. Given that SPs presented simple, textbook presentations of medical cases, that did not require lengthy physical examinations, this amount of time should have been sufficient to correctly manage a patient. Hence, unlike some settings, where the duration of consultations seems so short that it is unlikely that providers could offer high quality care – for instance, less than four minutes in a study in India (Das et al., 2012) – consultations seem to be sufficiently long in the study setting. Therefore, consultation duration does not seem to be a bottleneck for quality. The production possibility frontier does not highlight how many hours would be needed to achieve high quality care, but rather how many consultations can be fitted in a 5-hour working day
