# Supplementary material for: Does high workload reduce the quality of healthcare? Evidence from rural Senegal
Source: J Health Econ. 2022 Mar;82:102600. doi: 10.1016/j.jhealeco.2022.102600 (PMC9023795; doi:10.1016/j.jhealeco.2022.102600)
Supplement: Supplementary file 1 [file mmc1.docx]

**Online Appendix
Does high workload reduce the quality of healthcare? Evidence from rural Senegal**

[A.1 The standardised patient method 3](#_Toc94956440)

[A.1.1 Clinical cases 3](#_Toc94956441)

[A.1.2. Recommended history questions and physical examinations 4](#_Toc94956442)

[Dysentery 4](#_Toc94956443)

[Stable angina 5](#_Toc94956444)

[Family planning 6](#_Toc94956445)

[Asthma 7](#_Toc94956446)

[Tuberculosis 8](#_Toc94956447)

[A.1.3 Harmful and unnecessary drugs and treatments 9](#_Toc94956448)

[A.1.4 SP scripts 10](#_Toc94956449)

[Dysentery 10](#_Toc94956450)

[Stable angina 12](#_Toc94956451)

[Family planning 14](#_Toc94956452)

[Asthma 17](#_Toc94956453)

[Tuberculosis 20](#_Toc94956454)

[A.2. Additional tables 22](#_Toc94956455)

[Table A1 – Facilities and providers seen during busy and quite times 22](#_Toc94956456)

[Table A2 – Patient load, provider effort and the quality of case management (quadratic term) 23](#_Toc94956457)

[Table A3 – Facility busyness (continuous) and quality of healthcare 24](#_Toc94956458)

[Table A4 – Facility busyness (quintiles) and quality of healthcare 25](#_Toc94956459)

[Table A5 – Facility busyness (deciles) and quality of healthcare 26](#_Toc94956460)

[Table A6 – Facility busyness (alternative thresholds) and quality of healthcare 27](#_Toc94956461)

[Table A7 – Differences in quality of care and provider effort between professional cadres and other staff 28](#_Toc94956462)

[A.3. Instrumental Variable approach 28](#_Toc94956463)

[A.3.1 Motivation and validity 28](#_Toc94956464)

[Figure A1 – Patient load by day of the week 29](#_Toc94956465)

[Table A8 – Differences between Monday and other days 30](#_Toc94956466)

[Table A9 – Provider motivation based on day of the week of provider interview 31](#_Toc94956467)

[A.3.2 Results 31](#_Toc94956468)

[Table A10 – Patient load and day of the week (first-stage regression) 32](#_Toc94956469)

[Table A11 – Effect of workload on care quality (IV estimates) 32](#_Toc94956470)

[A.4. Robustness checks 33](#_Toc94956471)

[Table A12 – Caseload and care quality, with interactions by provider knowledge 34](#_Toc94956472)

[Table A13 – Patient load, busyness and care quality (excluding larger facilities) 35](#_Toc94956473)

[Table A14 – Patient load, busyness and care quality (SE clustered at provider level) 36](#_Toc94956474)

[Table A15 –Waiting times and care quality 37](#_Toc94956475)

[Table A16 – Patient load, busyness and care quality: heterogeneity between facilities with high and low average patient load 38](#_Toc94956476)

[References: 39](#_Toc94956477)

# A.1 The standardised patient method

## A.1.1 Clinical cases

The table below provides an overview of the five clinical cases used for the standardised patients, as well as recommended patient management according to local and international guidelines.

| **Clinical case** | **Description** | **Preferred case management** |
| --- | --- | --- |
| Dysentery | Child with bloody diarrhoea | ORS & Zinc & Metronidazole |
| Family planning | Woman asking for a contraceptive to space out births, history of migraines with aura | Any contraceptive, except hormonal methods |
| Asthma | Respiratory difficulty when coming in contact with dust | Salbutamol, corticoids or antihistamines |
| Stable angina | Pressing chest pain (radiating towards neck) following physical activity | Referral or ECG |
| Tuberculosis | Persistent cough, blood in sputum, weight loss | Screening for TB or referral |

## A.1.2. Recommended history questions and physical examinations

The list of relevant history questions and physical examinations included in the checklist shown below.

### Dysentery

Questions:

1. Did the child throw up?
2. Does the child eat normally?
3. Did the child have convulsions?
4. Is the child able to drink?
5. How many times a day does the child defecate?
6. What do the stools look like (watery, colour, etc.)?
7. Is there blood in the child’s stool?
8. Are there worms in the child’s stool?
9. Does the child have any other symptoms?
10. Did/does the child have fever?
11. Did you give the child any medication?
12. Do other people in the house also have diarrhoea?
13. Where does the water that the child drinks come from?
14. Do you boil the water before the child drinks it?
15. Do you wash your hands after going to the toilet or before feeding your child?
16. Is the colour of the child’s urine normal?
17. How many times a day does the child urinate?
18. Did the child already get a dose of Vitamin A?
19. Is the child fully vaccinated?

Physical examinations:

1. Measure height of the child
2. Weigh the child
3. Take temperature
4. Measure circumference of arm
5. Check the pulse
6. Check respiratory frequency
7. Check signs of dehydration (skin fold etc)
8. Check signs of anaemia (paleness of hands and feet)
9. Examine the abdomen of the child

### Stable angina

Questions:

1. How is the pain (stinging/ pressing)?
2. Was the pain very strong?
3. Where is the pain located?
4. Does the pain move around/ do you feel it at multiple places?
5. Did you lose consciousness?
6. What did you do when the pain started?
7. Did you have difficulties breathing/ were you short of breath?
8. Did the pain increase when you were breathing in?
9. Did you already have this kind of problem before?
10. Did you take any medication?
11. Did you have a headache?
12. Do you smoke?
13. Do you drink alcohol?
14. What is your diet like/ Do you eat a lot of fatty foods?
15. Do you engage in a regular physical activity?
16. Did someone in your family have similar problems?
17. Do you have diagnosed cardiac problems or problems with high blood ?pressure
18. Do you have diabetes?

Physical examinations:

1. Take temperature
2. Check pulse (rate, rhythm, volume)
3. Check blood pressure essential
4. Use stethoscope to listen to the heart
5. Use stethoscope to listen to the lugs(lungs)
6. Check if legs are swollen
7. Examine eyes
8. Check signs of anaemia (look at palms and nails)

### Family planning

Questions:

1. Age
2. The age of your last child/ your last birth?
3. How many pregnancies/ children you had?
4. Did you ever have a miscarriage?
5. Do you breast feed at the moment?
6. Are you using a contraceptive method at the moment?
7. When did you have your last period?
8. When did you last have intercourse?
9. Do you have any medical problems?
10. More details on the headaches (describe them, how is the pain? Where? In what situations do you have headaches?)
11. Do you have medical problems in your family?
12. Questions about your period (frequency, number of days, quantity, etc.)
13. Do you take any medication?
14. Do you consume coffee, alcohol or cigarettes?
15. Have you ever used a family planning method?
16. For how many years would you like not to have children?

Physical examinations:

1. Pregnancy test
2. Measure height
3. Weight
4. Take blood pressure
5. Examine eyes
6. Examine breasts
7. Examine abdomen
8. Examine legs
9. Examine thyroid

### Asthma

Questions:

1. What did you do when it happened/ What caused this episode/attack?
2. More details on what the patient felt during the episode (describe sensations)
3. How long did it take?
4. Was there a whistling noise?
5. Did you eat anything unusual?
6. Did you take any medication?
7. Did this happen before?
8. More details on the previous episode?
9. How often does this happen to you?
10. Did you also cough?
11. Was the cough dry or productive (with sputum)?
12. Did you have chest pain?
13. Did you have fever?
14. Do you have any other symptoms/ problems?
15. Did you have these types of problems (respiratory difficulties) in your childhood?
16. Did you take anything against these problems in your childhood?
17. Do you have any medical problems in your family?
18. Do you have cases of asthma or allergies in your family?
19. Do you have any known allergies?
20. Do you use incense or are you exposed to cigarette smoke?
21. Do you have a throat ache or a cold?

Physical examinations:

1. Take temperature
2. Take blood pressure
3. Measure respiratory frequency
4. Examine throat
5. Use stethoscope to listen to the heart
6. Use stethoscope to listen to the lugs

### Tuberculosis

Questions:

1. Do you have trouble breathing?
2. For how long exactly have you been coughing?
3. Is the cough productive/ is there sputum?
4. Is there blood in the sputum?
5. Do you have chest pain?
6. Do you cough all day long?
7. Does anybody in your surroundings have the same cough?
8. Did you have fever?
9. Do you have night sweats?
10. Do you have chills?
11. Did you lose weight?
12. Do you eat normally/ Do you have an appetite?
13. How do you feel in general?
14. Do other people in your family cough a lot or have asthma?
15. What is your job?
16. Do you have any other pains or symptoms?
17. Did you take any medication?
18. Do you use incense or are you exposed to cigarette smoke?

Physical examinations:

1. Take temperature
2. Take blood pressure
3. Take pulse
4. Take respiratory frequency
5. Use stethoscope at the front
6. Use stethoscope at the back
7. Do rapid diagnostic test for malaria

## A.1.3 Harmful and unnecessary drugs and treatments

| **SP case** | **Harmful drugs** | **Unnecessary drugs** |
| --- | --- | --- |
| Dysentery | opium tincture | analgesics, antipyretic, anthelminthic, antiseptic, antifungal, decongestant, expectorant, intestinal absorbent, probiotic, spasmolytic, treatment for malnutrition, vitamins |
| Angina | Nonsteroidal anti-inflammatory drug | amino acid, analgesics, antipyretic, antacid drugs, antibiotics, antiemetic, anthelminthic, antihistamines, antispasmodic, antituberculosis drug, anxiolytic, expectorant, diuretic, mucolytic, NSAID cream, proton pump inhibitor, steroids, supplements, vitamins |
| Asthma | Nonsteroidal anti-inflammatory drug | antacid drugs, anthelminthic, antibiotics, expectorant, opiate/opioids, analgesics, antipyretic, decongestant, diuretic, proton pump inhibitor supplements, vitamins, stimulants |
| Tuberculosis | - | anthelminthic, antihistamines, antibiotics, antimalarial, barbiturate, bronchodilator, cough suppressant, expectorant, opiate/opioids, NSAID, decongestant, haemostatic, supplements, vitamins, stimulants |
| Family planning | - | - |

## A.1.4 SP scripts

### Dysentery

1. **Personal History**

- **You and your children:**

As a mystery patient you always call yourself [your real name]; you are married ; you have XX children, including XX [name of last child], who is XX months [real age of child].

- **Why are you in the area:**

You live in one of the villages near the health post. You moved into the community a short time ago. You came to the in-laws to wait for your husband who has a mission abroad.

- **Appearance and attitude:**

You are of modest origin, and your dress code reflects this - you wear a boubou and sandals, and you do not wear makeup

1. **Medical History**

- **Your child's health in general**

Your child is generally healthy, apart from the small problems typical of children: sometimes a cough, a runny nose, a little fever or diarrhoea when teething. Your child usually has a good appetite and eats everything. You are careful to use mosquito nets to avoid malaria.

- **The problem that brings you**

Your son / daughter has had diarrhoea for 2 days now. He has had diarrhoea a few times in the past, but it usually goes away faster. You were in a bit of a hurry when you left and you did not find their health boklet.

You have enough to pay for the consultation ticket, but no money to buy the drugs; you will therefore take care to keep the prescription given to you if necessary

**In response to the provider who says "what brings you?"**

My son / daughter has had diarrhoea for 2 days now.

| **QUESTIONS** | **ANSWERS** |
| --- | --- |
| Has the child vomited? | No |
| Does the child eat normally? | He eats little |
| Has the child had seizures? | No, no seizures |
| Has the child (had) a fever? | No, I do not think so |
| Are you giving him a drink? Is the child able to drink? | Yes |
| What water do you give him? | Water from the well that my husband brought back |
| Do you boil this water? | No |
| How many bowel movements per day? | 4-5 bowel movements per day |
| How much does he do each time? | Quite a few each time |
| What do stools look like (type of stool: liquids, color, etc.)? | The stools are quite liquid and sometimes a little yellow |
| Is there blood in the stool? | Yes. I think I saw it twice |
| What is the color of the blood (in the stool)? | Red |
| Are there worms in the child's stool? | No |
| Does your child have any other symptoms? | I do not believe |
| Did you give him any medicine? | No |
| Is he still breastfed? | No |
| What did he eat? | As usual: rouy (porridge) and vegetables, rice and sometimes meat |
| Do other people in the house also have diarrhoea? | No |
| Do you wash your hands after using the bathroom or before feeding your child? | In general yes |
| If the urine colour is normal? | Yes |
| How many times does the child urinate per day? | As usual - about 3-4 times a day |
| When was the last time the child urinated? | 2-3 hours ago |
| Are his vaccinations up to date? | Yes |
| Was vitamin A given? | Yes |
| Do you sleep under a mosquito net? | Yes |
| Does the child play on the floor? | Yes. |
| Does the child put his dirty hands in his mouth? | Yeah, probably. |
| Does the child drink a lot? | He drinks what I give him. |
| Does the child cry when he has a bowel movement? | No. |
| Has the child been dewormed? | Yes |
| When did the deworming go back? | About 2 months |

### Stable angina

1. **Personal History**

- **You and your family:**

Your name is xxx [your real name]. You are married and have [XXX] children (as in reality). You have [XXX] brothers and [XXX] sisters (as in reality), they are in good health

Your parents died when you were approx. 30-35 years old:

- - Your father complained of chest pains much like the ones you had; he died of an infection
  - Your mother died after a fever
- **Why are you in the area:**

You have lived in the locality for not very long (less than 2 months) to get closer to your brother / friend to work in the fields

- **Appearance, attitude and personality:**

You like to eat well, especially rich dishes (e.g. tiebou yapp, mafé yapp, tiébou dieune, thiéré bassé yapp)

You drink palm wine regularly, especially with your friends.

You smoke almost 1 pack per day (around 15-20 cigarettes, Marlboro or Excellence and rolled tobacco - poon), and for a long time (around 20 years)

1. **Medical History**

- **Your health in general**

You are generally in good health, despite the fact that you smoke and eat a little too much! You have had a fever (malaria) a few times, but otherwise nothing else.

- **The problem that brings you**

Yesterday while you were riding your bike, you suddenly had a terrible pain in your chest, and it lasted about 5-10 min; the pain was very strong and pressing (diss) as if something heavy was pressing on you.

Some months ago you had felt the same kind of pain, also while you were on your bike carrying something heavy uphill (you had not gone to consult because the pain had passed quickly and you thought that you drank too much and ate too much).

**In response to the provider who says "what brings you?" ":**

Yesterday, I had pain in my chest and the pain was very strong. It had happened to me before.

| **QUESTIONS** | **ANSWERS** |
| --- | --- |
| Can you describe your pain to me?  What is the type / nature of the pain (acute / heavy / pressing)? | I felt like something very heavy was pressing me there (patient points to chest). |
| Was the pain very bad? | Yes. I stopped what I was doing and sat down until it passed |
| Where exactly is the pain? | There, towards the middle (shows the middle of the chest with the open hand, as if to press on the chest) |
| Does the pain radiate / be felt elsewhere? | Yes a little to the left side, and to the neck |
| Did he lose consciousness of you? | No |
| How long have you felt this pain? | About 10 minutes |
| What did you do to make it stop? | I sat down and waited for it to pass |
| What were you doing when the pain started? | I was riding a bicycle - it was 5 minutes |
| How often do you cycle? | Not often |
| Did you have difficulty breathing / were short of breath? | Yeah, kinda like I gasped in pain |
| Did the pain increase when breathing? | No |
| Have you ever had this kind of problem? | Only once more about 1 month ago |
| Do you remember what you were doing? | I was also riding a bicycle - had to stop when the pain took hold of me |
| Was the pain the same or different this time and yesterday? | It was pretty much the same |
| Why didn't you come to consult earlier? | I was far from home when it happened, and then it happened - I thought it was nothing at all, that I had drunk too much and ate too much. |
| Have you taken any medication for this pain? | No |
| Have you been nauseous or vomiting? | No |
| What did you eat? | As usual - meat, rice, Thiébou yapp, Thiéré bass yapp |
| Do you have digestion problems, diarrhoea, constipation? | No |
| Have you had a fever? | No |
| Did you have a headache? | No. |
| Do you smoke? | Yes - almost 1 pack per day (15-20 cigarettes) - malboro and also poon |
| Do you drink alcohol? | Yes, I like to drink palm wine (soum soum) with my friends |
| What is your diet / do you eat a lot of fatty things? | I like rich dishes - Tiébou yapp, mafé yapp, tiébou dieune, thiéré bassé yapp |
| Do you have a particular physical activity? | No not particularly |
| Does anyone in your family have / had a similar problem? | Yes my father sometimes had  this kind of pain I think |
| Is your father still alive? | No, he died when he was about 50 |
| How did he die? | We don't know exactly, he had an infection |
| What about your mother? | She also died a few years later, after a fever |
| Do you have a history of heart disease (blood pressure)? | I don't know No I don't think so |
| Do you have diabetes? | No |
| Are you taking any medication? | No |

### Family planning

1. **General personal history**

- **You and your family :**

Your name is [your name]. You are 27 years old.

You have been married for 7 years (monogamous marriage) - your husband is a mason and you have 4 children (12m, 2.5 years old, 4 years old and 5 years old) - 2 girls and 2 boys

You have brothers and sisters and in particular a 38-year-old sister who has just learned that she has breast cancer (she lives in Thiès).

Your parents are in good health and live near Dakar. Your mother is a little tired with heavy and very swollen legs.

- **Why are you in the area:**

You have lived in the village for about 1 month; your husband wanted to come for work

- **Appearance, attitude and personality:**

You are a shy and reserved person; you speak in a low voice and you are dressed modestly.

1. **Medical History**

- **Your health in general**

You are generally in good health, but you often have very severe headaches (since the age of about 16). This headache comes back quite often (every 2-3 weeks) the pain is very strong on one side.

Often before the headaches start you have like zigzag lines in front of your eyes (which remain even when you close your eyes usually these vision problems last 15-20 min). Sometimes you also vomit.

- **The problem that brings you**

You and your husband are very happy with the four children and now you want to wait a bit to have more children; one of your sisters told you about several planning methods

**In response to the provider who says "what brings you?" :**

I don't want to have other children before 2 years old, and I would like to know what methods are possible.

| **QUESTIONS** | **ANSWERS** |
| --- | --- |
| What is your age ? | 27 years old |
| Polygamous marriage? | No |
| At what age did you get married? | 20 years |
| Age of last child?  Date of last childbirth? | 12 months / It's been 1 year |
| How many kids do you have ? | 4 children (12m, 2.5 years old, 4 years old and 5 years old) |
| How many pregnancies have you had? | 4 pregnancies |
| Have you ever had a miscarriage | No |
| Have you had stillbirths? | No |
| Are you currently under method? | No |
| Have you used any methods before? | No |
| Are you exclusively breastfeeding? | No |
| Why don't you want more children? | Not at the moment - I would like to rest a little |
| Date of last period | My cycle has just ended |
| Questions about its cycle  Regularit? n large quantities ? | Regular cycle; My periods are very heavy and painful. |
| How many days does your period last? | 5 days |
| Have you had sex since you finished? | No  (The last report was just before menstruation) |
| Are there any medical problems in your family? | My mother (48 years old) often has very swollen legs which hurt her a lot.  I have a sister (who is 38 and lives in Thiès) (just learned that she) has breast cancer. |
| Do you have any specific medical problems? | In general, it's okay; but I often have very, very intense headaches. |
| Are you taking any medication for these headaches? | I take paracetamol but it doesn't work, it doesn't matter |
| Can you give me more details about these headaches? | They are very common (once every 2-3 weeks) and very strong on one side.  Before the headache starts I have like zigzag lines in front of my eyes for a while;  sometimes I also vomit and find it hard to bear the noise and the light |
| What do you do when this happens? | I go to bed and wait for it to pass - sometimes it lasts all day |
| Do the headaches come regularly / at times? | It happens a lot - sometimes just before your period, sometimes at other times |
| How old have you had this? | Since the age of 16 |
| Have you ever had an operation? | Never |
| Are you taking any medication? | No. |
| For how many years do you wish you had no children? | I'm not sure - maybe 2 years |
| Do you have tension? | No |
| Do you have diabetes? | No |
| At what age did you have your period? | [*give real age*] |
| What do you know about family planning? | Not much. My sister told me a bit about it. |
| What did your sister tell you? | She told me about the pill. But i don't know much |
| Which method do you want to use? | My sister told me the pill was good |
| Which pill do you want to use? | I do not know. |
| What is your profession ? | I am a housewife |
| What is your grade level? | Primary cycle |
| What is your husband's occupation? | He is a mason |
| Do you drink alcohol? | No |
| Do you smoke ? | No |
| Do you drink tea? | A little sometimes |
| Do you drink coffee? | No |
| Who told you about family planning? | My sister |
| Have you been referred from another health post? | No. |
| Do you have any specific complaints during your cycle? | Yes. My periods are very heavy and painful. |

### Asthma

1. **General Personal History**

- **You and your family :**

Your name is [your real name]. You are x years old [your actual age]. You have X brothers and X sisters [as in reality]. You are not married and you have no children yet

- **Why are you in the area:**

You are a student in Dakar but at the moment classes have not yet resumed so you are in the village next door with your aunt - your parents live in the town of the region (for example Tamba ville for Tambacounda).

- **Occupation and personality:**

You are following a hotel training course. You live in Djourbel with other students in a room in a student residence. You are a hard worker and don't go out a lot.

1. **Medical History**

- **Your health in general**

You don't drink, don't smoke; apart from the problem that brings you, you have no particular medical problem, but if the doctor asks you specific questions about your childhood, you remember that maybe when you were a child you had a little trouble breathing a few times - but you don't remember the circumstances well and it didn't last.

You are allergic to fish.

Everyone in your family is generally healthy, even if your mother has a lot of colds and red eyes during the dry season.

For about 2-3 months, you have had several times problems breathing.

If you are asked, you remember what happened when you had these issues:

- - Many times when you had a cold.
  - Two other times it happened after running - for example once to catch a bus.

You think the symptoms are getting worse and worse - this is how you might describe how you are feeling:

- - It is as if there is not enough air coming in
  - Sometimes there was like a whistling sound
  - It didn't last long (a few minutes)
  - You did not consult because you thought it was going to pass (has a cold, you do not do a lot of sport)
- **The problem that brings you**

Yesterday you had trouble breathing again, and it lasted longer than usual (almost 10 minutes) and you are worried

- - When it happened you were helping your aunt to clean the house and there was a lot of dust ; you had trouble catching your breath, then finally after 10 minutes maybe, it was better.
  - You felt very short of breath and could not catch your breath

**In response to the provider who says "what brings you?" :**

"The past few months I have had difficulty breathing at times. Yesterday again I couldn't find my breath for maybe 10 minutes."

| **QUESTIONS** | **ANSWERS** |
| --- | --- |
| What were you doing when it happened? | I was doing housework. Suddenly I couldn't really breathe. It's like I'm suffocating |
| How long did it last? | About 10 minutes I really had trouble breathing normally, then it got better but I felt tired |
| Was there something like a hissing noise ? | Yes |
| Did you eat anything different than usual? | No |
| Has this ever happened before? | Yes several times. |
| How many times has it happened | I'm not sure - 4.5 times? |
| What was the occasion / What were you doing when it happened before? | Twice it was after running,  Other times it was when I had a cold. |
| How many times does this happen to you in a month? | At first once or twice a month, now more often |
| Have you coughed too? | Yes I was coughing too |
| Was the cough productive or dry? | Cough is dry - no sputum / nothing coming out |
| Did you have pain in your chest? | No |
| Have you had a fever? | No |
| Have you had any other symptoms? | I had a cold and a runny nose for almost two weeks, but it's over now. |
| Did you have any similar problems (cough, difficulty breathing) in your childhood? | I remember when I was little several times I had trouble breathing |
| Did you take anything for these problems when you were younger? | Different things - syrups and also a little pump to breathe when it happened |
| Medical problems in the family? | No, no problem except maybe my mother: during the dry season, she has a runny nose and itchy eyes. |
| Is there asthma / similar breathing difficulties in the family? | No |
| Do you have any known allergies? | I am allergic to black fish |
| Have you used incense or been exposed to cigarette smoke? | I use incense sometimes |
| Are you allergic to frankincense? | No |
| Do you have a sore throat? | No |
| Do you have a sore throat or a cold? | I just had a cold for almost 2 weeks |
| Do you have asthma?  Have you ever been told you have asthma? | I don’t know  No |

### Tuberculosis

1. **General Personal History**

- **You and your family :**

Your name is [your real name]. You are [your true age]. You have X brothers and X sisters [as in reality]: You are married and you have x children.

- **Your job**

You work on construction sites in Dakar, as a mechanic; During your work sites, you live in a room with the other workers (8 per room).

You have just spent 2 months in Dakar working for a cousin who has a car repair business; you were replacing someone, so now you wait for the next job.

- **How you are in the locality:**

In the village, you stay with a cousin who you came to visit because the site you worked on in Dakar is over.

1. **Medical History**

- **Your health in general**

You do not drink or smoke and have not had any specific health problem in the past (apart from malaria a few times). Everyone in your family is generally healthy: parents still alive; overall in good health; siblings also healthy

- **The problem that brings you**

During the last 2 weeks, you haven't done much because you feel tired, you have very little appetite and you cough a lot; you have also been waking up for 1 week at night, sweating. You thought it would go away on its own so you didn't come to consult sooner; your wife told you to come because it was not going any better.

**Introductory sentence:**

I have been coughing for two weeks and I am not feeling well.

| **QUESTIONS** | **ANSWERS** |
| --- | --- |
| Are you having trouble breathing? | No. |
| How long have you been coughing? | 2 weeks |
| Is the cough dry or productive? | It's a fatty cough |
| Do you sometimes spit? | Yes |
| Is there blood in the sputum? | Yes |
| How is the sputum? | They are a little yellow. |
| Do you have chest pain? | Sometimes |
| Do you cough all day? | Yes, I cough a lot. |
| Is the cough worse in the morning? | Yes. When I wake up it's worse. |
| Do other people in your house have the same cough? | No. I don't think so. |
| Do you use incense or are exposed to cigarette smoke? | I do not smoke.  No incense at home. |
| Do you have had a fever? | Yes. A little I think. Overnight. |
| Do you have night sweats? | Yes, my sheets are sometimes wet when I get up. |
| Do you have chills? | Yes, sometimes a little, |
| Do you have lost weight? | Yes: my tight pants are a bit wide |
| How much do you weigh? | I do not know exactly |
| How much weight have you lost? | I don't know exactly - but my clothes are less tight |
| Do you have lost your appetite? | Yes, I eat very little and I'm not really hungry |
| Do you drink alcohol? | No |
| Do you smoke ? | No |
| How do you feel in general? | I feel very tired and weak |
| Is have people in your family cough a lot, have asthma? | No |
| What do you do as a job ? | I am a mechanic, I work on construction sites around Dakar. |
| Do you have other pain / problems ? | No - I feel very tired and weak |
| Have you taken any medication? | No. |
| Why didn't you come earlier? | I thought it was gonna go by on its own |
| Why are you coming now? | I really feel very tired and cannot work;  My wife and friends told me to come over |
| Did you consult someone (healer / other doctor)? | No |
| How old are your children ? | [as in reality] |
| How/where do you live? | In Dakar I share the room with 8 people  In the village I live with my family |
| Is where you live well ventilated? | In Dakar no; in the village yes, |

# A.2. Additional tables

### Table A1 – Facilities and providers seen during busy and quite times

|  |  | Busy | | Quiet | | p-val. |
| --- | --- | --- | --- | --- | --- | --- |
|  |  | Mean | SD | Mean | SD |  |
| **Panel A: Facility characteristics (N=195)** | | | | | | |
| Health post | | 0.88 | 0.33 | 0.96 | 0.19 | 0.02 |
| Time of the day the patient visit occurred: | |  |  |  |  |  |
| Before 10 am | | 0.43 | 0.5 | 0.51 | 0.50 | 0.30 |
| Between 10 am and 12 pm | | 0.41 | 0.49 | 0.37 | 0.48 | 0.54 |
| After 12 pm | | 0.16 | 0.37 | 0.12 | 0.33 | 0.53 |
| % of drugs and equipment available | | 0.79 | 0.09 | 0.78 | 0.08 | 0.63 |
| Number of facilities in 5km radius | | 1.66 | 3.08 | 1.29 | 2.18 | 0.33 |
| Distance to closest higher-level facility (km) | | 37.24 | 36.87 | 35.92 | 33.38 | 0.79 |
| % of treatment guidelines available | | 0.54 | 0.27 | 0.51 | 0.26 | 0.39 |
|  | |  |  |  |  |  |
| Observations | | 83 | | 112 | |  |
|  | |  | |  | |  |
| **Panel B: Provider characteristics (N=353)** | | | | | | |
| Male | | 0.52 | 0.5 | 0.46 | 0.5 | 0.24 |
| Work experience (years) | | 10.14 | 9.28 | 8.94 | 8.16 | 0.22 |
| Doctor | | 0.04 | 0.19 | 0.02 | 0.13 | 0.22 |
| Nurse | | 0.28 | 0.45 | 0.21 | 0.41 | 0.13 |
| Midwife | | 0.21 | 0.41 | 0.28 | 0.45 | 0.17 |
| Nursing assistant | | 0.26 | 0.44 | 0.31 | 0.46 | 0.33 |
| Other qualification | | 0.09 | 0.29 | 0.11 | 0.31 | 0.65 |
| No qualification | | 0.11 | 0.32 | 0.07 | 0.25 | 0.18 |
| Number of on-the-job training courses | | 2.97 | 2.82 | 2.37 | 2.46 | 0.04 |
| Provider born in local area | | 0.22 | 0.42 | 0.2 | 0.4 | 0.63 |
| Salary (in 1,000 FCFA) | | 140 | 84.75 | 131.93 | 84.22 | 0.42 |
|  | |  |  |  |  |  |
| Observations | | 107 | | 246 | |  |

Notes: In the analysis, busyness is measured at the SP consultation level (i.e. whether at the time SPs arrived at the facility, the number of patients waiting in line to see the same provider was higher than on an “average” day for that facility). This implies that some SP visits to the same facility (or with the same provider) were coded as “busy”, whilst other were not. For the purpose of this table, a facility is defined as “busy” if at least one of the SP consultations to the facility was coded as “busy”. Similarly, a provider is defined as “busy” if at least one of the SP consultations they conducted was coded as “busy”. SPs were seen by 364 providers but provider characteristics are available for only 353 of them. Data on salary are only available for n=343 providers and data on place of birth is only available for n=229 providers.

### Table A2 – Patient load, provider effort and the quality of case management (quadratic term)

|  | % relevant actions | | Duration | | Correct management | |
| --- | --- | --- | --- | --- | --- | --- |
|  | (1) | (2) | (3) | (4) | (5) | (6) |
|  |  |  |  |  |  |  |
| Patient load | 0.001 | 0.001 | -0.014 | -0.032 | 0.009 | 0.006 |
|  | (0.002) | (0.002) | (0.093) | (0.094) | (0.007) | (0.008) |
| Patient load X Patient load | -0.000 | -0.000 | -0.000 | 0.000 | -0.000 | -0.000 |
|  | (0.000) | (0.000) | (0.003) | (0.003) | (0.000) | (0.000) |
|  |  |  |  |  |  |  |
| Observations | 817 | 797 | 817 | 797 | 817 | 797 |
| R-squared | 0.532 | 0.539 | 0.542 | 0.552 | 0.317 | 0.329 |

Notes: All models show coefficients from linear (OLS) regressions The dependent variables are: (1-2) the proportion of relevant actions (history questions and physical examinations) done by providers, (3-4) the duration of the consultation in minutes and (5-6) the probability of correct case management. Dependent variables are regressed on patient load and patient load squared, where patient load is the number of patients waiting in line to be seen by the same provider when an SP arrived at the facility. All models control for region, district, facility, day-of-the-week, SP case and SP actor fixed effects, as well as the time of day (i.e. the hour) the visit occurred. Facility characteristics (facility type, availability of essential drugs and equipment, distance to closest higher-level facility, availability of essential treatment guidelines, number of facilities in 5km radius). Provider characteristics (gender, work experience, on-the-job training, level of education). Standard errors clustered at the provider level in parentheses. ***p<0.01, **p<0.05, * p<0.1

### Table A3 – Facility busyness (continuous) and quality of healthcare

|  | (1) | (2) | (3) |
| --- | --- | --- | --- |
|  | Correct case management | % relevant actions | Duration (minutes) |
| Facility busyness (continuous) | 0.002 | 0.004 | -0.231 |
|  | (0.033) | (0.008) | (0.425) |
|  |  |  |  |
| Facility and provider controls | Yes | Yes | Yes |
|  |  |  |  |
| Observations | 796 | 796 | 796 |
| R-squared | 0.326 | 0.537 | 0.552 |

Notes: All models show coefficients from linear (OLS) regressions. The dependent variables are: (1) the proportion of relevant actions (history questions and physical examinations) done by providers, (2) the duration of the consultation in minutes and (3) the probability of correct case management. Busyness is a continuous variable, defined at the SP consultation level, which is equal to the number of patients already waiting in line at the facility to see the same provider when SPs arrived (${PL}_{ipf}$), divided by the number of patients seen per provider at that facility on an “average” day ($\bar{PL}_{f})$. Facility characteristics (facility type, availability of essential drugs and equipment, distance to closest higher-level facility, availability of essential treatment guidelines, number of facilities in 5km radius). Provider characteristics (gender, work experience, on-the-job training, level of education). All models control for region, district, facility, day-of-the-week, SP case and SP actor fixed effects, as well as the time of day (i.e. the hour) the visit occurred. Standard errors clustered at the provider level in parentheses. ***p<0.01, **p<0.05, * p<0.1

### Table A4 – Facility busyness (quintiles) and quality of healthcare

|  | (1) | (2) | (3) |
| --- | --- | --- | --- |
|  | Correct case management | % relevant actions | Duration (minutes) |
| Patient visits on 2^nd^ least busy quintile | 0.052 | 0.018 | 0.288 |
|  | (0.078) | (0.022) | (1.031) |
| Patient visits on median quintile | 0.024 | 0.010 | -0.209 |
|  | (0.077) | (0.024) | (1.000) |
| Patient visits on 2^nd^ busiest quintile | 0.128 | 0.024 | 0.800 |
|  | (0.086) | (0.024) | (1.036) |
| Patient visits on busiest quintile | -0.035 | 0.014 | -0.504 |
|  | (0.089) | (0.024) | (1.097) |
|  |  |  |  |
| Facility and provider controls | Yes | Yes | Yes |
|  |  |  |  |
| Observations | 796 | 796 | 796 |
| R-squared | 0.334 | 0.538 | 0.553 |

Notes: All models show coefficients from linear (OLS) regressions. The dependent variables are: the proportion of relevant actions (history questions and physical examinations) done by providers, the duration of the consultation in minutes and the probability of correct case management. Explanatory variables are binary indicators for busyness, measured in quintiles at the consultation level. Our continuous measure of busyness divides the number of patients already waiting in line at the facility to see the same provider when SPs arrived, by the number of patients seen per provider at that facility on an “average” day. Binary variables in the table above show whether a given SP consultation was in the second least busy, median, second most busy or busiest quintile in the sample. Facility characteristics (facility type, availability of essential drugs and equipment, distance to closest higher-level facility, availability of essential treatment guidelines, number of facilities in 5km radius). Provider characteristics (gender, work experience, on-the-job training, level of education). All models control for region, district, facility, day-of-the-week, SP case and SP actor fixed effects, as well as the time of day (i.e. the hour) the visit occurred. Standard errors clustered at the provider level in parentheses. ***p<0.01, **p<0.05, * p<0.1

### Table A5 – Facility busyness (deciles) and quality of healthcare

|  | (1) | (2) | (3) |
| --- | --- | --- | --- |
|  | Correct case management | % relevant actions | Duration (minutes) |
| 2^nd^ least busy decile | 0.020 | -0.040 | -1.424 |
|  | (0.127) | (0.038) | (1.701) |
| 3^rd^ decile | -0.050 | -0.014 | -1.535 |
|  | (0.110) | (0.031) | (1.126) |
| 4^th^ decile | 0.141 | 0.026 | 1.196 |
|  | (0.096) | (0.028) | (1.370) |
| 5^th^ decile | 0.057 | -0.004 | -0.139 |
|  | (0.092) | (0.028) | (1.273) |
| 6^th^ decile | 0.006 | 0.003 | -0.947 |
|  | (0.102) | (0.031) | (1.286) |
| 7^th^ decile | 0.110 | 0.020 | 1.633 |
|  | (0.107) | (0.029) | (1.370) |
| 8^th^ decile | 0.164 | 0.009 | -0.684 |
|  | (0.106) | (0.028) | (1.213) |
| 9^th^ decile | -0.059 | 0.016 | -0.345 |
|  | (0.106) | (0.030) | (1.288) |
| Busiest decile | 0.025 | -0.009 | -1.511 |
|  | (0.110) | (0.028) | (1.543) |
|  |  |  |  |
| Facility and provider controls | Yes | Yes | Yes |
|  |  |  |  |
| Observations | 796 | 796 | 796 |
| R-squared | 0.341 | 0.542 | 0.563 |

Notes: All models show coefficients from linear (OLS) regressions. The dependent variables are: (1) the proportion of relevant actions (history questions and physical examinations) done by providers, (2) the duration of the consultation in minutes and (3) the probability of correct case management. Explanatory variables are dummy variables for busyness levels, measured in deciles based on a continuous measure of busyness (the ratio of the number of patients waiting in line at the facility when a SP arrives over the number of patients seen per provider at that facility on an “average” day). Facility characteristics (facility type, availability of essential drugs and equipment, distance to closest higher-level facility, availability of essential treatment guidelines, number of facilities in 5km radius). Provider characteristics (gender, work experience, on-the-job training, level of education). All models control for region, district, facility, day-of-the-week, SP case and SP actor fixed effects, as well as the time of day (i.e. the hour) the visit occurred. Standard errors clustered at the provider level in parentheses. ***p<0.01, **p<0.05, * p<0.1

### Table A6 – Facility busyness (alternative thresholds) and quality of healthcare

|  | (1) | (2) | (3) |
| --- | --- | --- | --- |
|  | Correct case management | % relevant actions | Duration (minutes) |
|  |  |  |  |
| Busy time (1.25x average) | -0.037 | -0.004 | -1.330 |
|  | (0.064) | (0.019) | (0.945) |
|  |  |  |  |
| Busy time (1.5x average) | -0.009 | -0.017 | -1.984** |
|  | (0.083) | (0.021) | (1.041) |
|  |  |  |  |
| Busy time (1.75x average) | -0.020 | -0.022 | -1.533 |
|  | (0.092) | (0.023) | (1.347) |
|  |  |  |  |
| Busy time (2x average) | 0.015 | -0.028 | -1.088 |
|  | (0.089) | (0.026) | (1.804) |
|  |  |  |  |
| Busy time (3x average) | 0.098 | 0.042 | -0.584 |
|  | (0.129) | (0.035) | (1.702) |
|  |  |  |  |
| Observations (consultations) | 796 | 796 | 796 |

Notes: Each cell represents the result of one OLS specification where the dependent variable indicated on the top row is regressed on a series of controls and a variable indicating how busy the facility was when the SP consultation occurred. The table reports results for five different binary measures of busyness, where a moment is defined as busy when the queue for a provider when the SP arrived (${PL}_{ipf}$) was 1.25 times, 1.5 times, 1.75 times, 2 times or 3 times larger than the number of patients seen per provider at that facility on an “average” day ($\bar{PL}_{f})$. The dependent variables are: (1) the proportion of relevant actions (history questions and physical examinations) done by providers, (2) the duration of the consultation in minutes and (3) the probability of correct case management. Facility characteristics (facility type, availability of essential drugs and equipment, distance to closest higher-level facility, availability of essential treatment guidelines, number of facilities in 5km radius). Provider characteristics (gender, work experience, on-the-job training, level of education). All models show coefficients from linear (OLS) regressions. All models control for region, district, facility, day-of-the-week, SP case and SP actor fixed effects, as well as the time of day (i.e. the hour) the visit occurred. Standard errors clustered at the provider level in parentheses. ***p<0.01, **p<0.05, * p<0.1

### Table A7 – Differences in quality of care and provider effort between professional cadres and other staff

|  |  | Professional cadres | | Other staff | | p-val. |
| --- | --- | --- | --- | --- | --- | --- |
|  |  | Mean | SD | Mean | SD |  |
| % relevant actions | | 0.32 | 0.16 | 0.29 | 0.15 | 0.01 |
| Duration (minutes) | | 13.25 | 8.17 | 12.05 | 7.27 | 0.03 |
| Correct management | | 0.38 | 0.49 | 0.33 | 0.47 | 0.11 |
|  | |  |  |  |  |  |
| Observations | | 393 | | 404 | |  |

Notes: The table shows the difference in the quality of care provided, as well as provider effort, between professional cadres (doctors, nurses and midwives) and other staff (with lower levels of qualification or not qualification).

# A.3. Instrumental Variable approach

## A.3.1 Motivation and validity

As there might be measurement error in the way we capture busyness (incomplete consultations registers or SPs miscounting the number of patients who attend), we use an instrument, which captures whether a given consultation occurred on a Monday. There are two main reasons why there are likely more patients attending facilities on Mondays. First, primary care facilities in Senegal generally operate Monday to Friday, meaning that for patients who fall ill over the weekend, Monday is the earliest opportunity to seek care. Second, in study areas, village markets often take place on Mondays. Individuals who travel to villages to attend markets often also attend health facilities on the same day to avoid multiple trips.

Figure A1 plots patient load by day of the week where patient load was 0.30 SD higher than on other days (6.3 compared to 4.6 patients, p<0.001). The proportion of consultations done on each day of the week is balanced and equal to approximately 20% for each day of the week. As shown in Table A7, we find that providers and facilities seen on Mondays as supposed to other days do not differ based on observables – which we take as support for the independence assumption.

### Figure A1 – Patient load by day of the week

Note: The average patient load (number of patients waiting to be seen when SPs arrived), and its 95% confidence interval, is shown by day of the week for all 817 consultations undertaken by SPs. The proportion of consultations done is balanced by day of the week, as 20% (approximately n=164) of consultations were conducted on each day. The seven consultations conducted on Saturdays are not shown.

### Table A8 – Differences between Monday and other days

|  |  | Monday | | Other days | | p-val. |
| --- | --- | --- | --- | --- | --- | --- |
|  |  | Mean | SD | Mean | SD |  |
| **Panel A: Facility characteristics (N=195)** | | | | | | |
| Health post | | 0.94 | 0.24 | 0.92 | 0.27 | 0.59 |
| % of drugs and equipment available | | 0.78 | 0.09 | 0.78 | 0.08 | 0.75 |
| Number of facilities in 5km radius | | 1.25 | 1.73 | 1.6 | 3.09 | 0.36 |
| Distance to closest higher-level facility (km) | | 39.3 | 38.09 | 34.39 | 32.2 | 0.33 |
| % of treatment guidelines available | | 0.52 | 0.26 | 0.53 | 0.27 | 0.76 |
|  | | 83 | | 112 | |  |
| **Panel B: Provider characteristics (N=353)** | | | | | | |
| Male | | 0.49 | 0.5 | 0.47 | 0.5 | 0.71 |
| Work experience (years) | | 9.9 | 9.5 | 9.04 | 8.06 | 0.38 |
| Doctor | | 0.01 | 0.1 | 0.03 | 0.17 | 0.26 |
| Nurse | | 0.25 | 0.44 | 0.22 | 0.42 | 0.54 |
| Midwife | | 0.22 | 0.42 | 0.28 | 0.45 | 0.24 |
| Nursing assistant | | 0.31 | 0.47 | 0.29 | 0.45 | 0.64 |
| Other qualification | | 0.10 | 0.30 | 0.11 | 0.31 | 0.90 |
| No qualification | | 0.10 | 0.30 | 0.07 | 0.26 | 0.37 |
| Number of on the job training courses | | 2.35 | 2.42 | 2.64 | 2.66 | 0.34 |
| Provider born in local area | | 0.19 | 0.40 | 0.21 | 0.41 | 0.79 |
| Salary (in 1,000 FCFA) | | 129.57 | 77.17 | 136.51 | 87.35 | 0.48 |
|  | | 86 | | 267 | |  |

Notes: In the analysis we focus on whether a given SP consultation was conducted on a Monday. As SPs visited facilities on two consecutive days, it is possible that SPs visited the same facility (or the same provider) on a Monday, as well as another day of the week. For the purpose of this table, we focus on facilities and providers that had at least one SP consultation on a Monday. SPs were seen by 364 providers. Data on general provider characteristics are available for 353 of these providers. Data on salary are only available for n=343 providers and data on place of birth is only available for n=229 providers.

Another requirement for a valid instrument is that it affects the outcome only through the specified channel (i.e. the exclusion restriction). In our setting, the day of the week should not influence the quality of care through any other channel than workload. One might argue that there is a plausible alternative channel – provider motivation. It is possible that, the quality of care is systematically different on Mondays, as providers are more or less motivated (as they either find it challenging to settle into work after the weekend, or are more relaxed due to their time off).

We are able to test for whether providers’ motivation differs on Mondays by using data from the health worker interview (conducted prior to SP visits). We examine providers’ self-reported intention to quit their current job and the degree to which they agree with various statements about their work satisfaction, including, “I am happy with the variety of tasks I have to do”, “I am happy with my workload”, “My salary is satisfactory” and “I like my working environment”, differs for providers interviewed on Mondays as opposed to other days. As shown in Table A9, we find no evidence to suggest that providers who were interviewed on Mondays differ significantly in terms of motivation. However, two statements are close to being significantly different and higher on Mondays "There is sufficient medicine available for me to work" and "I like my work environment." Hence, we cannot rule out that there are some differences in terms of provider motivation on Mondays and other days, which could be potentially suggestive of more systemic, unobservable, differences influencing behaviours beyond the issue of workload – which would violate the exclusion restriction.

### Table A9 – Provider motivation based on day of the week of provider interview

|  |  | Monday | | Other days | | p-val. |
| --- | --- | --- | --- | --- | --- | --- |
|  |  | Mean | SD | Mean | SD |  |
| I intend to quit my current job | | 0.38 | 0.49 | 0.27 | 0.45 | 0.19 |
| I like the imagine people have of my profession | | 0.93 | 0.27 | 0.94 | 0.23 | 0.63 |
| I am happy with the variety of tasks I have to do | | 0.93 | 0.27 | 0.93 | 0.25 | 0.84 |
| I am unhappy with my workload | | 0.40 | 0.50 | 0.46 | 0.50 | 0.46 |
| There is sufficient medicine available for me to work | | 0.53 | 0.51 | 0.39 | 0.49 | 0.12 |
| My salary is satisfactory given the work I provide | | 0.10 | 0.30 | 0.13 | 0.34 | 0.58 |
| I am satisfied with my contract | | 0.45 | 0.50 | 0.50 | 0.50 | 0.59 |
| I am happy with the distribution of workload | | 0.8 | 0.41 | 0.79 | 0.41 | 0.89 |
| I like my work environment | | 0.65 | 0.48 | 0.50 | 0.50 | 0.09 |
| My tasks correspond well to my skills | | 0.93 | 0.27 | 0.92 | 0.27 | 0.96 |
| Colleagues appreciate my work | | 0.90 | 0.30 | 0.90 | 0.30 | 0.99 |
| Satisfied with work schedule | | 0.35 | 0.48 | 0.44 | 0.50 | 0.29 |
| Satisfied with level of responsibility entrusted in me | | 0.95 | 0.22 | 0.94 | 0.23 | 0.90 |
|  | |  |  |  |  |  |
| Observations | | 40 |  | 181 |  |  |

Notes: The table shows the proportion of health providers who agree or strongly agree with the statements shown based on the day of the week the provider interview was conducted.

We estimate the following models:

| ${Patient load}_{ipf}=\alpha_{0}+\alpha_{1}{Monday}_{i}+\alpha_{2}X_{p}+\alpha_{3}Z_{f}++\delta_{c}+\delta_{a}+\delta_{r}+\delta_{d}+v_{ipf}$ | *(1)* |
| --- | --- |
| $Y_{ipf}=\beta_{0}+\beta_{1}\hat{{Patient load}_{ipf}}+{{\beta_{2}X}_{p}+\beta_{3}Z}_{f}++\delta_{c}+\delta_{a}+\delta_{r}+\delta_{d}+e_{ipf}$ | *(2)* |

In the first stage (equation 1) we regress patient load on the instrument (${Monday}_{i}$).

In the second stage (equation 2) we regress quality of care ($Y_{ipf}$) on the predicted values of patient load from equation 1.

## A.3.2 Results

As shown in Table A10 (the first stage), we find that patient load is systematically higher on Mondays. The Cragg-Donald F-statistic is 7.58.

We use Stock and Yogo’s approach to test for weak instruments, which suggests that Monday is indeed a weak instrument (Lee et al., 2020) – meaning that results should be interpreted with caution.

Table A11 shows the second stage. We find no evidence the workload affects provider effort or the quality of case management. These estimates are quite precise and close to zero.

### Table A10 – Patient load and day of the week (first-stage regression)

|  | (1) |
| --- | --- |
|  | Patient load |
|  |  |
| Monday | 1.141** |
|  | (0.544) |
|  |  |
| Cragg-Donald minimum eigenvalue | 7.58 |
| Stock and Yogo critical value 10% | 16.38 |
| Stock and Yogo critical value 15% | 8.96 |
| Observations | 797 |
| R-squared | 0.414 |

Notes: We control for region, district, SP case and SP fixed effects as well as time of day of the visit, facility and provider characteristics. Standard errors clustered at the provider level in parentheses. ***p<0.01, **p<0.05, * p<0.1.

### Table A11 – Effect of workload on care quality (IV estimates)

|  | (1) | (2) | (3) |
| --- | --- | --- | --- |
|  | % relevant actions | Duration | Correct management |
|  |  |  |  |
| Patient load (instrumented) | -0.004 | 0.333 | -0.023 |
|  | (0.011) | (0.577) | (0.036) |
|  |  |  |  |
| Clusters (providers) | 353 | 353 | 353 |
| Observations (consultations) | 797 | 797 | 797 |

Notes: All models show coefficients from linear (OLS) regressions. All models control for region, district, SP case and SP fixed effects, as well as the time of day the visit occurred. The dependent variables are: the proportion of relevant actions (history questions and physical examinations) done by providers, the duration of the consultation in minutes and the propagability of correct case management. All models control for facility characteristics (facility type, availability of essential drugs and equipment, distance to closest higher-level facility, availability of essential treatment guidelines, number of facilities in 5km radius) and provider characteristics (gender, work experience, on-the-job training, level of education). Standard errors clustered at the provider level in parentheses. ***p<0.01, **p<0.05, * p<0.1.

# A.4. Robustness checks

We use clinical vignettes – a role-playing exercise where providers are asked to diagnose hypothetical patients – to measure providers’ clinical knowledge (as in Das *et al.*, 2015; Mohanan *et al.*, 2015; Kovacs, *et al*., 2020). In the role-play, an enumerator acting as a hypothetical patient began the ‘consultation’ by reading out an introductory statement to describe their primary symptom (for example “I have been coughing for two weeks”). Providers were able to ask for further information and ‘perform’ physical examinations or tests – receiving answers to questions and examination/test results based on a script. Each role-play concluded with the provider indicating how they would manage the patient. Another enumerator observed the role-play and noted down the providers’ actions (questions asked, physical examinations and tests ‘performed’, drugs and treatments recommended).

Depending on the types of patients seen by providers in the past six months (children, adults, and/or family planning) providers completed up to five clinical vignettes in a random order. Vignettes were designed to precisely match SP cases (dysentery, family planning, asthma, angina and tuberculosis). Hypothetical patients reported the same symptoms as the SPs and responded in the same way to providers’ questioning. We generate a binary measure of provider knowledge that indicates whether case management was correct in vignettes – using the same criteria applied to SP data.

### Table A12 – Caseload and care quality, with interactions by provider knowledge

|  | (1) | (2) | (3) |
| --- | --- | --- | --- |
|  | Correct case management | % relevant actions | Duration (minutes) |
| **Panel A: Patient load** | | | |
| Patient load | -0.009 | 0.000 | -0.162 |
|  | (0.009) | (0.002) | (0.114) |
| Provider knows how to treat SP | 0.050 | 0.066** | 0.404 |
|  | (0.097) | (0.026) | (1.253) |
| Patient load * Provider knows | 0.002 | -0.001 | 0.138 |
|  | (0.010) | (0.002) | (0.128) |
| Observations | 498 | 498 | 498 |
| R-squared | 0.417 | 0.639 | 0.667 |
|  |  |  |  |
| **Panel B: Busyness (quasi-random variation)** | | | |
| Busy time | -0.190 | -0.005 | -2.366 |
|  | (0.104) | (0.034) | (1.426) |
| Provider knows how to treat SP | 0.038 | 0.054** | 0.758 |
|  | (0.093) | (0.024) | (1.212) |
| Busy time * Provider knows | 0.047 | 0.024 | 0.865 |
|  | (0.152) | (0.040) | (1.946) |
| Observations | 498 | 498 | 498 |
| R-squared | 0.423 | 0.639 | 0.668 |

Notes: All models show coefficients from linear (OLS) regressions. The dependent variables are: (1) the proportion of relevant actions (history questions and physical examinations) done by providers, (2) the duration of the consultation in minutes and (3) the probability of correct case management. In Panel A, dependent variables are regressed on patient load, which is the number of patients waiting in line to be seen by the same provider when an SP arrived at the facility. In Panel B, dependent variables are regressed on a dummy variable indicating whether the number of patients waiting to be seen when the SP arrived at the facility exceeded the number of patients seen on an “average” day. We lack data on busyness for one SP consultation, as data on “average” consultation volumes could not be collected in the facility where this visit occurred. All models control for region, district, facility, day-of-the-week, SP case and SP actor fixed effects, as well as the time of day (i.e. the hour) the visit occurred. Facility characteristics (facility type, availability of essential drugs and equipment, distance to closest higher-level facility, availability of essential treatment guidelines, number of facilities in 5km radius). Provider characteristics (gender, work experience, on-the-job training, level of education). Standard errors clustered at the provider level in parentheses. ***p<0.01, **p<0.05, * p<0.1

### Table A13 – Patient load, busyness and care quality (excluding larger facilities)

|  | % relevant actions | | Duration | | Correct management | |  |
| --- | --- | --- | --- | --- | --- | --- | --- |
|  | (1) | (2) | (3) | (4) | (5) | (6) |  |
| **Panel A: Patient load** | | | | | | | |
| Patient load | -0.001 | -0.000 | 0.013 | 0.014 | 0.004 | 0.001 |  |
|  | (0.002) | (0.002) | (0.075) | (0.077) | (0.006) | (0.006) |  |
|  |  |  |  |  |  |  |  |
| Facility and provider controls | No | Yes | No | Yes | No | Yes |  |
|  |  |  |  |  |  |  |  |
| Observations (consultations) | 758 | 743 | 758 | 743 | 758 | 743 |  |
| R-squared | 0.527 | 0.535 | 0.540 | 0.551 | 0.320 | 0.335 |  |
|  |  |  |  |  |  |  |  |
| **Panel B: Busyness (quasi-random variation)** | | | | | | |  |
| Busy time | -0.005 | -0.002 | -1.557 | -1.606 | -0.093 | -0.109 |  |
|  | (0.018) | (0.019) | (0.892) | (0.911) | (0.068) | (0.072) |  |
|  |  |  |  |  |  |  |  |
| Facility and provider controls | No | Yes | No | Yes | No | Yes |  |
|  |  |  |  |  |  |  |  |
| Observations (consultations) | 757 | 742 | 757 | 742 | 757 | 742 |  |
| R-squared | 0.527 | 0.534 | 0.543 | 0.554 | 0.320 | 0.337 |  |

Notes: All models show coefficients from linear (OLS) regressions. The larger healthcare facilities (i.e. health centres) are excluded from the analytical sample. The dependent variables are: (1-2) the proportion of relevant actions (history questions and physical examinations) done by providers, (3-4) the duration of the consultation in minutes and (5-6) the probability of correct case management. In Panel A, dependent variables are regressed on patient load, which is the number of patients waiting in line to be seen by the same provider when an SP arrived at the facility (equation 1). In Panel B, dependent variables are regressed on a dummy variable indicating whether the number of patients waiting to be seen when the SP arrived at the facility exceeded the number of patients seen on an “average” day (equation 2). We lack data on busyness for one SP consultation, as data on “average” consultation volumes could not be collected in the facility where this visit occurred. All models control for region, district, facility, day-of-the-week, SP case and SP actor fixed effects, as well as the time of day (i.e. the hour) the visit occurred. Facility characteristics (availability of essential drugs and equipment, distance to closest higher-level facility, availability of essential treatment guidelines, number of facilities in 5km radius). Provider characteristics (gender, work experience, on-the-job training, level of education). Standard errors clustered at the provider level in parentheses. ***p<0.01, **p<0.05, * p<0.1

### Table A14 – Patient load, busyness and care quality (SE clustered at provider level)

|  | % relevant actions | | Duration | | Correct management | |
| --- | --- | --- | --- | --- | --- | --- |
|  | (1) | (2) | (3) | (4) | (5) | (6) |
| **Panel A: Patient load** | |  |  |  |  |  |
| Patient load | -0.001 | -0.001 | -0.019 | -0.030 | 0.003 | 0.001 |
|  | (0.001) | (0.002) | (0.068) | (0.072) | (0.005) | (0.006) |
|  |  |  |  |  |  |  |
| Facility and provider controls | No | Yes | No | Yes | No | Yes |
|  |  |  |  |  |  |  |
| Clusters (providers) | 364 | 353 | 364 | 353 | 364 | 353 |
| Observations (consultations) | 817 | 797 | 817 | 797 | 817 | 797 |
| R-squared | 0.530 | 0.537 | 0.542 | 0.552 | 0.315 | 0.328 |
| **Panel B: Busyness (quasi-random variation)** | | |  |  |  |  |
| Busy time | -0.005 | -0.001 | -1.433 | -1.538 | -0.095 | -0.107 |
|  | (0.017) | (0.018) | (0.790) | (0.821) | (0.063) | (0.067) |
|  |  |  |  |  |  |  |
| Facility and provider controls | No | Yes | No | Yes | No | Yes |
|  |  |  |  |  |  |  |
| Clusters (providers) | 363 | 352 | 363 | 352 | 363 | 352 |
| Observations (consultations) | 816 | 796 | 816 | 796 | 816 | 796 |
| R-squared | 0.529 | 0.537 | 0.544 | 0.554 | 0.316 | 0.330 |
| Outcome mean | 0.308 | | 12.619 | | 0.351 | |

Notes: All models show coefficients from linear (OLS) regressions. The dependent variables are: (1-2) the proportion of relevant actions (history questions and physical examinations) done by providers, (3-4) the duration of the consultation in minutes and (5-6) the probability of correct case management. In Panel A, dependent variables are regressed on patient load, which is the number of patients waiting in line to be seen by the same provider when an SP arrived at the facility (equation 1). In Panel B, dependent variables are regressed on a dummy variable indicating whether the number of patients waiting to be seen when the SP arrived at the facility exceeded the number of patients seen on an “average” day (equation 2). We lack data on busyness for one SP consultation, as data on “average” consultation volumes could not be collected in the facility where this visit occurred. All models control for region, district, facility, day-of-the-week, SP case and SP actor fixed effects, as well as the time of day (i.e. the hour) the visit occurred. Facility characteristics (facility type, availability of essential drugs and equipment, distance to closest higher-level facility, availability of essential treatment guidelines, number of facilities in 5km radius). Provider characteristics (gender, work experience, on-the-job training, level of education). Standard errors clustered at the provider level in parentheses. ***p<0.01, **p<0.05, * p<0.1

### Table A15 –Waiting times and care quality

|  | % relevant actions | | Duration | | Correct management | |
| --- | --- | --- | --- | --- | --- | --- |
|  | (1) | (2) | (3) | (4) | (5) | (6) |
| Waiting times (minutes) | 0.000 | 0.000 | 0.003 | 0.000 | 0.001** | 0.001 |
|  | (0.000) | (0.000) | (0.007) | (0.008) | (0.000) | (0.000) |
|  |  |  |  |  |  |  |
| Facility and provider controls | No | Yes | No | Yes | No | Yes |
|  |  |  |  |  |  |  |
| Observations (consultations) | 817 | 797 | 817 | 797 | 817 | 797 |
| R-squared | 0.530 | 0.538 | 0.542 | 0.552 | 0.320 | 0.331 |

Notes: All models show coefficients from linear (OLS) regressions. All models control for region, district, facility, day-of-the-week, SP case and SP actor fixed effects, as well as the time of day (i.e. the hour) the visit occurred. The dependent variables are: (1-2) the proportion of relevant actions (history questions and physical examinations) done by providers, (3-4) the duration of the consultation in minutes and (5-6) the probability of correct case management. Facility characteristics (facility type, availability of essential drugs and equipment, distance to closest higher-level facility, availability of essential treatment guidelines, number of facilities in 5km radius). Provider characteristics (gender, work experience, on-the-job training, level of education). Standard errors clustered at the health facility level in parentheses. ***p<0.01, **p<0.05, * p<0.1

### Table A16 – Patient load, busyness and care quality: heterogeneity between facilities with high and low average patient load

|  | % relevant actions | | Duration | | Correct management | |  |
| --- | --- | --- | --- | --- | --- | --- | --- |
|  | High patient load | Low patient load | High patient load | Low patient load | High patient load | Low patient load |  |
|  | (1) | (2) | (3) | (4) | (5) | (6) |  |
| **Panel A: Patient load** | | | | | | |  |
| Patient load | -0.003 | 0.001 | -0.120 | -0.031 | -0.001 | 0.007 |  |
|  | (0.002) | (0.002) | (0.109) | (0.112) | (0.007) | (0.008) |  |
|  |  |  |  |  |  |  |  |
| Facility and provider controls | Yes | Yes | Yes | Yes | Yes | Yes |  |
|  |  |  |  |  |  |  |  |
| Observations (consultations) | 203 | 594 | 203 | 594 | 203 | 594 |  |
| R-squared | 0.690 | 0.533 | 0.627 | 0.573 | 0.573 | 0.348 |  |
|  |  |  |  |  |  |  |  |
| **Panel B: Busyness (quasi-random variation)** | | | | | | | |
| Busy time | -0.019 | 0.001 | -0.467 | -1.708 | -0.059 | -0.103 |  |
|  | (0.053) | (0.020) | (2.170) | (0.944) | (0.220) | (0.068) |  |
|  |  |  |  |  |  |  |  |
| Facility and provider controls | Yes | Yes | Yes | Yes | Yes | Yes |  |
|  |  |  |  |  |  |  |  |
| Observations (consultations) | 202 | 594 | 202 | 594 | 202 | 594 |  |
| R-squared | 0.682 | 0.533 | 0.622 | 0.576 | 0.570 | 0.351 |  |

Notes: All models show coefficients from linear (OLS) regressions. The table presents some sperate analysis for facilities that have a high patient load (defined as being in the top 75^th^ percentile of average patient load according to facility records), or low average patient load (below the 75^th^ percentile). The dependent variables are: (1-2) the proportion of relevant actions (history questions and physical examinations) done by providers, (3-4) the duration of the consultation in minutes and (5-6) the probability of correct case management. In Panel A, dependent variables are regressed on patient load, which is the number of patients waiting in line to be seen by the same provider when an SP arrived at the facility (equation 1). In Panel B, dependent variables are regressed on a dummy variable indicating whether the number of patients waiting to be seen when the SP arrived at the facility exceeded the number of patients seen on an “average” day (equation 2). We lack data on busyness for one SP consultation, as data on “average” consultation volumes could not be collected in the facility where this visit occurred. All models control for region, district, facility, day-of-the-week, SP case and SP actor fixed effects, as well as the time of day (i.e. the hour) the visit occurred. Facility characteristics (facility type, availability of essential drugs and equipment, distance to closest higher-level facility, availability of essential treatment guidelines, number of facilities in 5km radius). Provider characteristics (gender, work experience, on-the-job training, level of education). Standard errors clustered at the health facility level in parentheses. ***p<0.01, **p<0.05, * p<0.1

# References:

Das, J., Kwan, A., Daniels, B., Satyanarayana, S., Subbaraman, R., Bergkvist, S., Das, R.K., Das, V., Pai, M., 2015. Use of standardised patients to assess quality of tuberculosis care: A pilot, cross-sectional study. Lancet Infect. Dis. 15, 1305–1313. https://doi.org/10.1016/S1473-3099(15)00077-8

Kovacs, R.J., Lagarde, M., Cairns, J., 2020. Overconfident health workers provide lower quality healthcare. J. Econ. Psychol. 76, 102213. https://doi.org/10.1016/j.joep.2019.102213

Lee, D.S., McCrary, J., Moreira, M.J., Porter, J., 2020. Valid t-ratio Inference for IV. https://doi.org/10.3386/w29124

Mohanan, M., Vera-Hernández, M., Das, V., Giardili, S., Goldhaber-Fiebert, J.D., Rabin, T.L., Raj, S.S., Schwartz, J.I., Seth, A., 2015. The know-do gap in quality of health care for childhood diarrhea and pneumonia in rural India. JAMA Pediatr. 169, 349–57. https://doi.org/10.1001/jamapediatrics.2014.3445
